# Epigenetic landscape, key transcriptional regulators, and in vivo identification of human Tr1 cells

**DOI:** 10.1126/sciadv.aec6358

**Published:** 2026-07-10

**Authors:** Alma-Martina Cepika, Laura Amaya, Colin Waichler, Mansi Narula, Michelle Mantilla, Benjamin C. Thomas, Pauline P. Chen, Robert A. Freeborn, Mara Pavel-Dinu, Jason Nideffer, Matthew Porteus, Rosa Bacchetta, Fabian Müller, William J. Greenleaf, Howard Y. Chang, Maria Grazia Roncarolo

**Affiliations:** ^1^ Division of General Surgery, Department of Surgery, Stanford University School of Medicine, Stanford, CA, USA.; ^2^ Division of Hematology, Oncology, Stem Cell Transplantation and Regenerative Medicine, Department of Pediatrics, Stanford University School of Medicine, Stanford, CA, USA.; ^3^ Center for Definitive and Curative Medicine, Stanford University School of Medicine, Stanford, CA, USA.; ^4^ Institute for Stem Cell Biology and Regenerative Medicine, Stanford University School of Medicine, Stanford, CA, USA.; ^5^ Department of Dermatology, Stanford University School of Medicine, Stanford, CA, USA.; ^6^ Division of Immunology, Department of Pediatrics, University of Washington School of Medicine, Seattle, WA, USA.; ^7^ Division of Infectious Diseases and Geographic Medicine, Stanford University School of Medicine, Stanford, CA, USA.; ^8^ Integrative Cellular Biology and Bioinformatics, Saarland University, Saarbrücken, Germany.; ^9^ Department of Genetics, Stanford University School of Medicine, Stanford, CA, USA.; ^10^ Center for Personal Dynamic Regulome, Stanford University School of Medicine, Stanford, CA, USA.; ^11^ Howard Hughes Medical Institute, Stanford University, Stanford, CA, USA.

## Abstract

Type 1 regulatory T (Tr1) cells are CD4^+^ T cells with suppressive function that are induced from conventional T cells exposed to persistent or strong antigens. Human Tr1 cells are understudied; the regulators of their antigen-driven differentiation are unknown, and identifying them in tissues, where antigen interactions occur, is challenging. Here, we conducted a multiomic profiling of human antigen-induced Tr1 cells. Using CRISPR-based functional genomics, we uncovered essential roles of transcription factors IRF4, BATF, and MAF in human Tr1 differentiation, phenotype, and function. We also derived a Tr1 transcriptional signature that detects cells with a Tr1 phenotype in single-cell datasets from patients treated with Tr1 therapy and those with solid tumors. Cross-species analysis confirmed this signature identifies bona fide Tr1 cells induced in vivo in a murine solid tumor model. These findings provide a framework for development of Tr1-based and Tr1-targeting therapies and studies of Tr1 cell biology.

## Teaser

Multiomics approach reveals transcriptional regulators of human Tr1 cells and a gene signature to identify them in vivo.

## INTRODUCTION

Regulatory T cells (T_reg_ cells) play a critical role in human health by inducing and maintaining tolerance to endogenous and exogenous antigens (*1*). Unlike the T_reg_ cells that express transcription factor (TF) FOXP3 and arise as a distinct lineage of CD4^+^ T cells in the thymus, FOXP3^−^ type 1 regulatory T (Tr1) cells arise from peripheral, mature conventional CD4^+^ T cells in response to antigen stimulation in a tolerogenic environment, e.g., in the presence of immunoregulatory cytokines interleukin-10 (IL-10) or IL-27 (*2*). In murine models, Tr1 cells can be induced from either naïve or already polarized, memory CD4^+^ T helper (T_H_) cells, such as T_H_1, T_H_17, or T follicular helper cells (T_FH_ cells) (*2*, *3*), which could explain why a single defining TF that controls their differentiation has not been identified yet. Regardless of their ontogeny, Tr1 cells have potent suppressive function in vitro and in vivo (*2*, *4*, *5*), which is comparable to that of FOXP3^+^ T_reg_ cells (*6*) and mediated by IL-10, CTLA-4, and PD-1/PD-L1 pathway (*7*). Tr1 cells also express interferon-γ (IFN-γ), perforin, and granzymes (*7*) and suppress effector T cells (T_eff _ cell) by killing myeloid antigen-presenting cells (APC) (*8*–*10*) required for T_eff _ cell activation. In humans, Tr1 cells have been identified in antigen-driven chronic inflammatory conditions, such as allogeneic hematopoietic stem cell transplantation (allo-HSCT) (*11*–*13*), allergy (to bee venom, pollen, and dust mites) (*14*–*16*), infections (leishmania, malaria, and influenza) (*17*–*20*), and hematological malignancies (*21*), where they appear to play a beneficial role in subsiding inflammation.

However, data from solid tumors indicate that Tr1 cells suppress antitumor immunity. In humans, EOMES^+^ Tr1-like cells were found clonally expanded in primary and metastatic colorectal cancer (CRC) and non–small cell lung cancer (NSCLC) samples, suggesting that Tr1 cells were induced and/or expanded intratumorally (*22*). In a murine sarcoma model, neoantigen-induced Tr1 cells mediated resistance to anti-PD1 treatment by killing intratumoral APC and inhibiting adoptively transferred T_eff _ cells to reject the tumor (*23*). Both studies suggested that the presence of Tr1 cells correlates with worse outcomes in solid tumors, including CRC, NSCLC, and melanoma (*22*, *23*). These important studies show that there is more than one T_reg_ cell subtype that can interfere with antitumor immunity and that cancer immunotherapies designed to remove just FOXP3^+^ T_reg_ cells, e.g., by anti-CCR8 antibodies (*24*), may not be effective in tumors that also contain Tr1 cells. Thus, it is imperative that we identify Tr1 cells in human tumor tissue and understand their differentiation.

The coexpression of surface proteins CD49b and LAG3 can identify Tr1 cells among human peripheral blood CD4^+^ T cells and in vitro–induced Tr1 cells (*5*, *7*, *25*), but these markers have not been successful in identifying human Tr1 cells in the tissue (*22*, *26*). In addition, there is lack of consensus on TFs that govern human Tr1 cell differentiation, which is IL-10–driven (*27*) and likely not superimposable with IL-27–induced murine Tr1 ontogeny (*28*–*31*). Here, we combined epigenetic and transcriptional profiling to reveal the landscape of transcriptional regulators of human antigen-inducible Tr1 cells, followed by functional genomics to identify the role of *IRF4*, *BATF*, and *MAF* TFs in Tr1 differentiation and function. In addition, we reveal a transcriptional signature that can uncover cells with Tr1 features among circulating and tumor-resident human CD4^+^ T cells and identify bona fide murine Tr1 cells induced in vivo in response to neoantigens.

## RESULTS

### Human antigen-induced Tr1 cells have a unique epigenetic landscape

**Fig. 1.**
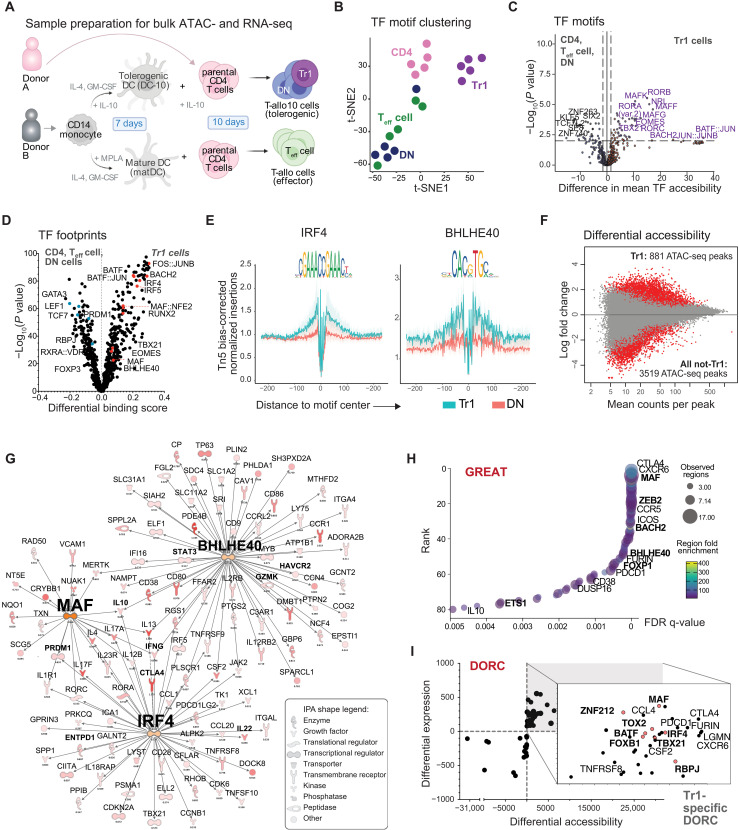
ATAC-seq reveals the chromatin accessibility landscape and transcriptional regulators of antigen-induced Tr1 cells. (**A**) Experimental outline, bulk ATAC- and RNA-seq. Tr1 = CD49b^+^LAG3^+^ CD45RA^−^CD4^+^ Tr1 cells from T-allo10 cell product; DN = double-negative (non-Tr1) CD49b^−^LAG3^−^ CD45RA^−^CD4^+^ cells from T-allo10 product; T_eff_ cell = CD49b^−^LAG3^−^ CD45RA^−^CD4^+^ effector T cells from control T-allo product; DC-10 = allogeneic tolerogenic dendritic cells; matDC = allogeneic mature dendritic cells; MPLA = monophosphoryl lipid A, a TLR4 agonist added to mature the dendritic cell (DC) at day 5. (**B**) tSNE visualization of ATAC-seq profiles using 452 TF motif deviations identified by chromVAR. Colored by cell type. (**C**) TF enrichment (chromVAR) between Tr1 and all non-Tr1 cells; *P* values from two-tailed *t* test. (**D**) Differential binding activity (TOBIAS) between Tr1 and all non-Tr1 cells; each dot represents one motif. Representative Tr1-specific TFs are highlighted in red, and non-Tr1 TFs in blue. (**E**) Bias-corrected ATAC-seq footprints between Tr1 and DN (non-Tr1) T-allo10 cells. Selected motifs with differential binding activity between Tr1 and non-Tr1 cells (D) are shown. (**F**) Chromatin accessibility differences between Tr1 cells (n = 5) and non-Tr1 cells (CD4, T_eff_ cell, and DN; total, *n* = 15). Each dot represents one ATAC-seq peak. In red: significantly different peaks. (**G**) Upstream regulator Ingenuity Pathway Analysis (IPA) of the most accessible peaks in Tr1 cells as ranked by GSEA, displaying a gene network associated with the top three transcriptional regulators with a rank of ≥0.7. (**H**) Regulatory element scores predicted by GREAT, ranked by chromatin accessibility. Cutoff = FDR *q* value < 0.005. Representative hub genes are indicated, with TFs in bold font. (**I**) Paired correlation between differential expression and differential accessibility; domains of regulatory chromatin (DORC), FigR. Enlarged top right quadrant shows Tr1-specific DORC and top associated genes (regular font, black dots) and TFs (bold, red dots).

We investigated the transcriptional regulation of Tr1 cell identity by analyzing in vitro antigen-induced Tr1 cells with assay for transposase-accessible chromatin using sequencing [ATAC-seq; (*32*)] and RNA sequencing (RNA-seq). We obtained Tr1 cells from T-allo10 cell products, made by coculturing peripheral blood CD4^+^ T cells with allogeneic tolerogenic dendritic cells (called DC-10) in the presence of IL-10 (*7*, *27*). Previously, we have shown that T-allo10 Tr1 cells are clonally expanded and have alloantigen-specific suppressive function (*7*), and T-allo10 cell products are being tested in an ongoing phase 1/1b trial (*7*, *33*) (ClinicalTrial.gov ID: NCT04640987). Here, we again purified Tr1 cells by fluorescence-activated cell sorting (FACS) based on CD49b and LAG3 coexpression in memory (CD45RA^−^) CD4^+^ T cells from five T-allo10 products (Fig. 1A) and compared their epigenome and transcriptome to three populations of control cells: (i) total parental CD4^+^ T cells (ex vivo isolated from peripheral blood); (ii) memory CD49b^−^LAG3^−^ [double-negative (DN)] cells purified from T-allo10 products (non-Tr1 cell controls exposed to IL-10 and DC-10 in vitro); and (iii) memory CD49b^−^LAG3^−^ T_eff _ cells purified from T-allo products. T-allo cells were induced from same parental CD4^+^ T cells but in coculture with mature instead of tolerogenic dendritic cells, resulting in enrichment of clonally expanded T_eff _ cells (Fig. 1A) (*7*). After quality control (fig. S1, A to C), we analyzed TF motif accessibility in Tr1 cells versus control cell populations using ChromVar (*34*) and showed that Tr1 cells cluster apart from other populations (Fig. 1B). TF motifs that were significantly more accessible in Tr1 cells included AP-1 (Fos, Jun, and others), MAF, IRF, and T-box TF family members (Fig. 1C). Footprinting analysis using TOBIAS (*35*) was concordant with the motif analysis, revealing many of the same TF family members active in Tr1 cells (Fig. 1D). TFs with prominent footprints, suggesting their active use in the cell at the time of analysis, included *IRF4*, which we previously identified as up-regulated in Tr1 cells (*7*), and *BHLHE40*, for which we have shown that it regulates IFN-γ and IL-10 production in Tr1 cells (*25*) (Fig. 1E).

Next, we compared the chromatin accessibility of Tr1 cells versus control populations, which revealed 881 significantly accessible peaks in Tr1 cells (Fig. 1F). We annotated the peaks based on their nearest gene and analyzed the list of genes accessible in Tr1 cells using Gene Set Enrichment Analysis (GSEA) (*36*) and the Molecular Signature Database (MsigDB) gene set C7: Immune Signatures (*37*). GSEA analysis showed that genes accessible in Tr1 cells were significantly enriched in MsigDB signatures of T_reg_ cells and memory T cells (fig. S1D). The same MsigDB signatures were also enriched in Tr1 cells in our published bulk RNA-seq analysis of Tr1 cells (*7*), showing reproducibility across independent datasets and platforms. We also ranked the accessible Tr1 genes based on a differential accessibility score with GSEA and used the ranked list to predict key transcriptional regulators of genes accessible in Tr1 cells with Ingenuity Pathway Analysis (IPA) (*38*) upstream regulator module. IPA analysis identified *IRF4*, *MAF*, and *BHLHE40* TFs as the top three transcriptional regulators of Tr1 cells (Fig. 1G and table S1).

Chromatin peaks that were significantly accessible in Tr1 cells compared to control populations overlapped with known *IRF4*, *MAF*, and *BHLHE40* promoter and enhancer regions (fig. S1E). To systematically infer regulatory elements such as enhancers for genes that were accessible in Tr1 cells, we used GREAT (*39*). Some of the most accessible regulatory elements in Tr1 cells were found adjacent to TF genes *MAF*, *BHLHE40*, and *ZEB2* and previously identified Tr1 genes *CTLA4*, *CXCR6*, *ICOS*, *FURIN*, *PDCD1*, *IL10* (*7*), and *CCR5* (*26*) (Fig. 1H). *MAF* regulates production of IL-10, an archetypal Tr1 cell cytokine (*40*), and *ZEB2* was recently shown to induce *GZMA* (encoding granzyme A) (*41*), which we found highly expressed in Tr1 cells (*7*).

To identify TFs that were both epigenetically active and transcribed in Tr1 cells, we integrated ATAC-seq data with matched RNA-seq data (Fig. 1A) using FigR (*42*). FigR identifies regions of high-density peak-gene associations known as domains of regulatory chromatin (DORC), which contain lineage-determining genes and overlap with known super-enhancers (*42*, *43*). Similarly to GREAT analysis, DORCs identified in Tr1 cells (table S2) encompassed archetypal Tr1 genes such as *CTLA4*, *PDCD1*, *CXCR6*, and *FURIN*, and TFs *MAF*, *IRF4*, *BATF*, *TBX21* (encoding T-bet), *TOX2*, *RBPJ*, *FOXB1*, *and ZNF212* (Fig. 1I and fig. S1F).

In summary, these data show that antigen-induced differentiation of human Tr1 cells leads to significant chromatin remodeling, with an emergence of a distinct pattern of accessible chromatin peaks, TF motifs, and footprints. In addition to *BHLHE40*, which function in Tr1 cells we have described previously (*25*), *MAF* and *IRF4* emerged as the likely transcriptional regulators of Tr1 cell identity in several analyses of gene regulation.

### Human antigen-induced Tr1 cells exhibit a distinct transcriptional profile and oligoclonal TCR repertoire at the single-cell level

**Fig. 2.**
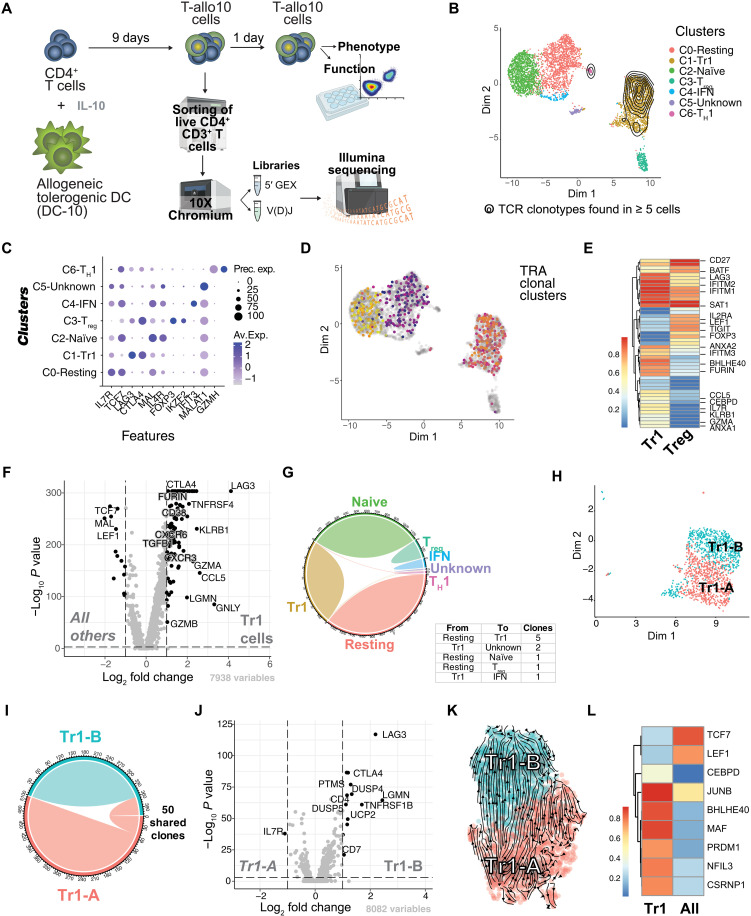
scRNA + TCR-seq analysis identifies antigen-induced Tr1 cells as a distinct population within T-allo10 cells. (**A**) Experimental outline, sc-immune profiling (sc-RNA + TCR-seq) of T-allo10 products. (**B**) UMAP visualization of T cell subpopulations within T-allo10 cells (*n* = 2). Overlaid contour plot shows clonally expanded cells. The lowest clonal frequency displayed is five cells. (**C**) Dot plot illustrating the representative differentially expressed genes (DEGs) per cluster. Dot color intensity corresponds to the average expression level per cluster. Dot size represents the percentage of cells expressing the gene within the cluster. (**D**) UMAP visualization of TRA clonal clusters; cells with similar CDR3 sequences by edit distance are represented with the same color. Cells in gray mean no identified clusters. (**E**) Heatmap of average expression of 49 DEGs identified between Tr1 and FOXP3^+^ T_reg_ cell clusters. Adjusted *P* (*P*adj) value ≤ 0.001 and log_2_ fold change (log_2_FC) ≥ 1. (**F**) Volcano plot depicting DEGs between Tr1 and all other clusters. DEG that passed the filtering from (E) are in black; representative genes are labeled. (**G**) Left: Chord diagram showing clonotypes shared between clusters. Each arc represents a UMAP cluster from (A), and ribbons connecting arcs represent shared clonotypes. The width of the ribbons corresponds to the number of shared clonotypes. Right: Table summarizing the clonotypes shared between clusters. (**H**) UMAP projection displaying the reclustering of the Tr1 cluster from (A). (**I**) Chord diagram showing the number of unique and shared clones among Tr1 subclusters. (**J**) Volcano plot illustrating the differential gene expression analysis between Tr1 subclusters. DEG [filter as in (E)] in black**.** (**K**) Developmental trajectory of Tr1 cells predicted by RNA velocity. (**L**) Heatmap representing the average expression of 9 TFs differentially expressed between Tr1 cells and all other clusters. DC, dendritic cell.

Next, we sought to validate our findings at the single-cell level. We analyzed two T-allo10 cell products using single-cell (sc) immune profiling [5′ scRNA-seq and T cell receptor sequencing (TCR-seq)], simultaneously capturing the transcriptome and TCR repertoire (Fig. 2A). This approach allowed us to identify cells with Tr1 features that underwent clonal expansion in response to antigen stimulation during in vitro differentiation. The expected Tr1 cell phenotype and function was confirmed in the T-allo10 cell products at the end (day 10) of the T-allo10 culture (fig. S2, A to C), while an aliquot of T-allo10 cells at day 9 was purified for live CD4^+^CD3^+^ T cells by FACS before single-cell capture, library preparation, and sequencing.

After quality control (table S3) and filtering to remove doublets and dead cells, 4508 T-allo10 cells separated into seven clusters in a Uniform Manifold Approximation and Projection (UMAP) embedding (Fig. 2B and fig. S3A), which we annotated using data-driven (*44*) and knowledge-based methods (Fig. 2C and fig. S3, B to D). Cluster C1 was identified as Tr1 cells based on the expression of Tr1 markers *LAG3* and *ITGA2* (encoding CD49b), cytokines *IL10* and *IFNG*, and genes we previously identified in Tr1 cells such as *CTLA4*, *FURIN*, *GZMA*, and *CXCR6* (fig. S3, C and D). The proportion of cells in the Tr1 cluster C1 (average of 29.64%) at day 9 of Tr1 in vitro differentiation was similar to the proportion of Tr1 cells identified by flow cytometry at the end of culture (day 10, average of 36.05%; figs. S2B and S3A). As expected from antigen-induced cells (*7*), 31.56% of all clones were in the Tr1 cluster (Fig. 2B and fig. S3E). The other cluster with high number of clones was the small T_H_1 cluster C6 (fig. S3E). The clustering of the TCR complementarity-determining region 3 (CDR3) sequences by Levenshtein distance (*45*) showed that cells in the Tr1 cluster are clonally similar (Fig. 2D and fig. S3F). CDR3 clusters identified among Tr1 cells were predominantly contained within Tr1 cell cluster, with a few similar CDR3 sequences found in resting memory CD4^+^ T cells, and just one in T_reg_ cells (fig. S3G). These data support antigen-driven Tr1 clonal expansion during their in vitro differentiation from CD4^+^ T cells.

Next, we compared the transcriptome of Tr1 cells to other CD4^+^ T cells with overlapping functions: suppressive FOXP3^+^ T_reg_ cells and cytotoxic T_H_1 cells. The comparison of FOXP3^+^ T_reg_ cells and Tr1 cells identified 49 differentially expressed genes (DEGs) after stringent filtering [false discovery rate (FDR)–adjusted *P* value (*P*_adj_) < 0.001 and absolute log_2_ fold change (FC) ≥ 1; Fig. 2E and table S4]. FOXP3^+^ T_reg_ cells expressed high level of *FOXP3*, *IL2RA* (encoding CD25), *TIGIT*, *CD27*, and TF *BATF*. Compared to FOXP3^+^ T_reg_ cells, Tr1 cells expressed higher *LAG3*; IFN-regulated IFIT gene family (*IFITM1*, *IFITM2*, and *IFITM3*); *FURIN* (a protease which can activate latent transforming growth factor-β); genes conferring the cytotoxic phenotype (*KLRB1* and *GZMA*); chemokine *CCL5*; *IL7R* (encoding IL-7 receptor α-subunit); and TFs *BHLHE40*, *NFIL3*, and *CEBPD* (Fig. 2E). Compared to T_H_1 cells, Tr1 cells expressed higher levels of *LAG3*, *LGMN*, *CTLA4*, *FURIN*, and *MAF*, while T_H_1 cells expressed higher levels of *GZMH*, *FCRL6*, *FGFBP2*, *NKG7*, and *PRF1* (fig. S3H).

Tr1 cells exhibited a distinct transcriptional profile compared to all other clusters. Ninety genes were significantly up-regulated in the Tr1 cluster C1, including archetypal Tr1 genes associated with suppressive activity (*LAG3*, *CTLA4*, *FURIN*, and *TGFB1*) and cytotoxicity (*KLRB1*, *GZMA*, *GZMB*, *GNLY*, and *CCL5*) (Fig. 2F and table S5). We observed low expression of *EOMES* (fig. S4A), which was previously described as a marker of human polyclonal Tr1 cells and tissue Tr1 cells (*22*, *26*, *46*), and a positive regulator of proteins associated with cytotoxic function (perforin, granzyme B, and IFN-γ) in cytotoxic T cells (*47*). Despite the sparse expression of *EOMES*, the Tr1 cells expressed granzyme B protein (encoded by *GZMB*) and were able to release cytotoxic granules in response to stimulation (fig. S4, B and C).

Concordantly with the clonal cluster analysis, TCR repertoire analysis showed that Tr1 cells shared only eight clones with other clusters, five clones with cluster C0 (resting), two clones with cluster C5 (unknown), and one clone with cluster C4 (IFN; a cluster of T cells expressing IFN-regulated genes); no clones were shared with T_reg_ cells (Fig. 2G). Notably, FOXP3^+^ T_reg_ cells did not clonally expand in response to alloantigen stimulus (Fig. 2B and fig. S3F), likely because this dataset did not capture rare alloreactive FOXP3^+^ T_reg_ cells (*48*).

To assess Tr1 cell heterogeneity, we reclustered Tr1 cells, revealing two subclusters: Tr1-A and Tr1-B (Fig. 2H). The Tr1 subclusters shared 50 clones among 237 cells (Fig. 2I) and were phenotypically similar, with only 14 DEGs (Fig. 2J). Tr1 cells in cluster Tr1-B expressed higher levels of activation-inducible genes such as *LAG3*, *CTLA4*, and dual-specificity phosphatases (*DUSP4* and *DUSP5*), while Tr1 cells in cluster A had higher IL-7 receptor (IL-7R) (Fig. 2J and table S6), a hallmark of polarized memory CD4^+^ T cells (*49*) that is also up-regulated in the resting C0 cluster (Fig. 2D). RNA velocity analysis (*50*) suggested that the resting phenotype in cluster Tr1-A precedes a more activated phenotype in cluster Tr1-B (Fig. 2K). Only one TF, *JUNB*, was differentially expressed between these two clusters (2.24-fold higher in Tr1-B; adjusted *P* value = 6.24 × 10^−46^). Since *JUNB* is part of the AP-1 TF family, which is downstream of TCR signaling, its expression in the Tr1-B cluster is in accordance with its more activated state.

In comparison to all other T cell populations, Tr1 cells significantly up-regulated seven TFs: *CEBPD*, *JUNB*, *BHLHE40*, *MAF*, *PRDM1*, *NFIL3*, and *CSRNP1* (Fig. 2L). Besides *BHLHE40*, which regulates IFN-γ and IL-10 in Tr1 cells (*25*), some of these TFs have known functions in human T cells: *NFIL3* represses FOXP3 (*51*) and up-regulates *IL10* and *HAVCR2* (encoding Tim-3) (*52*), while *MAF* and *PRDM1* regulate IL-10 (*40*, *53*). The expression of *IRF4*, which was identified as one of the key Tr1 cell regulators in our epigenome analysis (Fig. 1), was high not only in Tr1 cells but also in FOXP3^+^ T_reg_ cell cluster (fig. S4D), indicating that *IRF4* could play a biological role in both regulatory T cell types.

Together, our sc-immune profiling analysis uncovered that human in vitro–differentiated, antigen-induced Tr1 cells have a distinct transcriptional signature, TCR repertoire, and TF expression profile. In line with our epigenome analysis, Tr1 cells expressed higher *MAF* and *BHLHE40* than other CD4^+^ T cell subsets, while *IRF4* was up-regulated in both Tr1 cells and FOXP3^+^ T_reg_ cells.

### Longitudinal single-cell multiome profiling of antigen-induced Tr1 cells reveals unique transcriptional programs and those shared with activated FOXP3^+^ cells

**Fig. 3.**
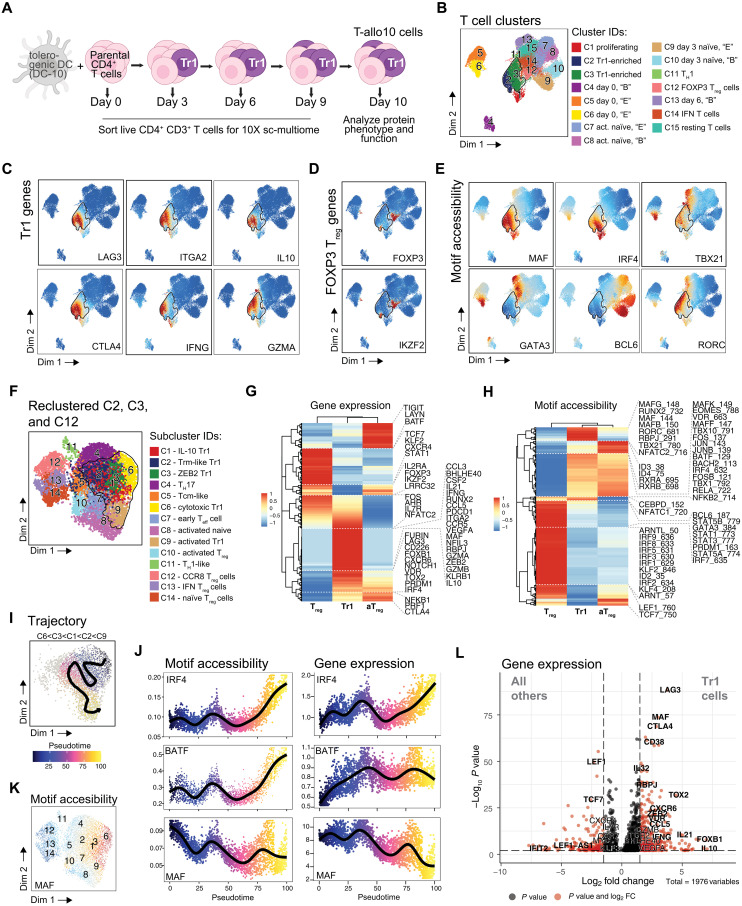
Longitudinal scRNA- + ATAC-seq analysis identifies distinct transcriptional and epigenetic landscape of antigen-induced Tr1 cells*.* (**A**) Experimental outline, longitudinal sc-multiome of T-allo10 cells. (**B**) UMAP visualization of sc-multiome data, with annotated clusters of CD4^+^ T cell populations. (**C**) UMAP embedding showing the expression of Tr1 marker genes. (**D**) UMAP embedding showing the expression of FOXP3^+^ T_reg_ cell marker genes. (**E**) Motif accessibility visualization of selected TFs; UMAP embedding. (**F**) Reclustering of Tr1-enriched and FOXP3^+^ T_reg_ cell populations from (B), with annotated subpopulations. (**G**) Heatmap showing differential expression of key marker genes distinguishing T_reg_ and Tr1 cells. (**H**) Heatmap of motif accessibility comparing TFs between T_reg_ and Tr1 cells. (**I**) Trajectory analysis aligning the indicated Tr1 cell clusters over pseudo-time axis. (**J**) Temporal dynamics of Tr1 marker expression and motif accessibility in Tr1 clusters over pseudo-time as in (I). (**K**) UMAP projection displaying *MAF* motif accessibility in clusters from (F). (**L**) Volcano plot depicting DEGs in Tr1 cells [clusters C1, C2, C3, C6, and C9 from (F)] compared to all other CD4^+^ T cells.

To understand the changes of the Tr1 epigenome during their antigen-induced differentiation, we analyzed T-allo10 cells longitudinally by sc-multiome (ATAC- and RNA-seq). Aliquots of purified live parental CD4^+^ T cells from two donors were analyzed before coculture with allogeneic DC-10 (day 0) and at days 3, 6, and 9 of T-allo10 culture (Fig. 3A). Expected phenotype and function of T-allo10 cell products were confirmed by flow cytometry at day 10 (end of culture; fig. S5). The sc-multiome analysis of 45,262 cells identified 15 distinct clusters (Fig. 3B and fig. S6A). In the UMAP embedding, parental (day 0) CD4^+^ T cell clusters from both donors separated from each other and from other cultured CD4^+^ T cells (days 3, 6, and 9) (fig. S6B). Cluster annotations were determined on the basis of representative gene expression profiles (fig. S6C and table S7). Archetypal Tr1 cell genes (*LAG3*, *ITGA2*, *IL10*, and *CTLA4*) were up-regulated in portions of clusters C2 and C3 (Fig. 3C), which were therefore labeled as Tr1 enriched. Genes associated with cytotoxic functions (*IFNG* and *GZMA*) were also expressed in Tr1-enriched clusters C2 and C3, in clusters of proliferating cells (C1), and in T_H_1 cells (C11; Fig. 3C). FOXP3^+^ T_reg_ cell genes, *FOXP3* and *IKZF2*, were expressed in a small fraction of cluster C2 and in the T_reg_ cell cluster C12 (Fig. 3D).

Most cells from Tr1-enriched clusters C2 and C3 were from days 6 and 9 of the culture (fig. S6B). RNA velocity analysis indicated that the cell states in cluster C3 were preceded by those in cluster C2 and cluster C1 (fig. S6D). Cluster C1 consisted of cells expressing cytotoxic and cell cycle-related genes, including *MKI67*, *TOP2A*, *CDK1*, *CDCA2*, *CDCA3*, and *CDC20* (fig. S6C and table S7). Since 29% of the cells in cluster C1 originated from day 3 of the culture (fig. S6B), it is possible that the cytotoxic, proliferating cells in this cluster represent precursors of Tr1 cells. Besides the expected Tr1-related genes *ITGA2*, *CTLA4*, and *BHLHE40*, the top dynamic genes in clusters C1, C2, and C3 included *NEAT1*, a long non-coding (lnc) RNA that suppresses T_H_17 differentiation (fig. S6D) (*54*).

*MAF* and *IRF4* motifs, which we identified in our bulk epigenome analyses (Fig. 1), were highly accessible in both Tr1-enriched clusters (C2 and C3) and in the proliferating cell cluster C1 (Fig. 3E). The motif of *TBX21* (encoding T-bet) had intermediate accessibility in clusters C1, C2, and C3; its accessibility was highest in the T_H_1 cluster C11 and in a subset of parental CD4^+^ T cells from donor “E” at day 0 (Fig. 3E). The accessibility of the *EOMES* motif paralleled the one of *TBX21* (fig. S6E). T_H_2 TF *GATA3* and T_FH_ cell TF *BCL6* were inaccessible in Tr1-enriched clusters C2 and C3, but T_H_17 TF *RORC* was highly accessible in a subset of cells from clusters C2 and C3 (Fig. 3E). The accessibility of *RORC*, along with the expression of *FOXP3* and *IKZF2* in a subset of cells from Tr1-enriched clusters C2 and C3, suggests that these Tr1-enriched clusters C2 and C3 contain other CD4^+^ T cell subtypes, such as T_H_17 cells and FOXP3^+^ T_reg_ cells (Fig. 3D).

To discriminate Tr1 cells from other transcriptionally and functionally similar CD4^+^ T cell populations, we increased the clustering resolution and reclustered Tr1-enriched clusters C2 and C3 and FOXP3^+^ T_reg_ cell cluster C12. Reclustering revealed 14 subclusters (Fig. 3F) with distinct donor and time-point distributions (fig. S6, F and G). These subclusters were annotated on the basis of their gene expression profiles (fig. S6H and table S8). Tr1 cells, identified by the elevated expression of *LAG3*, *ITGA2*, *CTLA4*, and *IL10* and the intermediate expression of cytotoxic genes such as *IFNG* were in subclusters C1, C2, C3, C6, and C9 (fig. S6I). T_H_17 cells separated into subcluster C4, marked by high *CCR6* expression (fig. S6H) and highest *RORC* expression (fig. S6I). FOXP3^+^ T_reg_ cells from the original cluster C12 (Fig. 3B) now separated into three subclusters (C14, C13, and C12), composed of naïve, IFN signature gene-expressing (IFN), and CCR8^HIGH^ T_reg_ cells, respectively. FOXP3^+^ cells that previously clustered with Tr1-enriched cells in cluster C2 now formed a separate subcluster C10, which had high expression of TCR-inducible genes *VAV3* and *LAYN*, the latter of which is characteristic for human activated and tissue-resident T_reg_ cells (*55*–*57*) (fig. S6H). Thus, cells in the C10 subcluster likely represent antigen-activated (a) T_reg_ cells.

As we did not observe cells corresponding to aT_reg_ cells (subcluster C10) in our sc-immune profiling analysis (Fig. 2), we compared the gene expression and motif accessibility of Tr1 cells (subclusters C1, C2, C3, C6, and C9 combined) to aT_reg_ cells and other FOXP3^+^ T_reg_ cells (subclusters C12, C13, and C14 combined) (Fig. 3F). Genes up-regulated only in Tr1 cells included TFs *BHLHE40*, *MAF*, *NFIL3*, *RBPJ*, *ZEB2*, and *RUNX2*; Tr1 marker genes *CCR5* (*26*), *ITGA2*, *PDCD1*, *CCL5*, *CCL3*, *GZMA*, *and GZMB*; and cytokine genes *IL10*, *IFNG*, *and IL21* (Fig. 3G). *MAF*, *RBPJ*, *TBX21*, and *RUNX2* TF motifs were accessible in Tr1 cells (Fig. 3H). Conversely, the expression of *FOXP3*, *IKZF2*, *IL2RA*, and *LRRC32* (encoding GARP) was restricted to aT_reg_ cells and FOXP3^+^ T_reg_ cells, while the up-regulation of *CTLA4*, *NFKB1*, and *PRF1* was observed in both Tr1 and aT_reg_ cells (Fig. 3G). The expression of Tr1 marker genes *LAG3*, *CD226*, *CXCR6*, and *FURIN* and TFs *IRF4*, *TOX2*, *PRDM1*, *FOXB1*, and *VDR* not only was highest in Tr1 cells but also observed in aT_reg_ cells (Fig. 3G). Similarly, Tr1 and aT_reg_ cells shared accessible TF motifs that were closed in FOXP3^+^ T_reg_ cells, which included *EOMES*, *BATF*, *VDR*, *IRF4*, and AP-1 and nuclear factor κB (NFκB) family TFs (Fig. 3H). Thus, some transcriptional and regulatory features are found in both Tr1 cells and aT_reg_ cells, while others are specific to Tr1 cells.

It was unexpected to observe that Tr1 cells had higher accessibility of *RORC*, a TF associated with T_H_17 cells, than FOXP3^+^ T_reg_ cells (Fig. 3H), because Tr1 cells produce no or very low IL-17 (*5*, *7*). The intermediate expression of *RORC* was also observed in cytotoxic, IFNG-expressing Tr1 cell subcluster, and in T_H_1-like cells (fig. S6I). Given that Tr1 cells exhibit more cytotoxic features earlier during their differentiation (fig. S7A), we hypothesized that *RORC* might activate early in Tr1 cell development, then down-regulate under the activity of T_H_17-repressing TFs *TBX21* and *EOMES* (*31*, *58*, *59*) and long noncoding RNA (lncRNA) *NEAT1* (fig. S6D). To test this, we conducted a trajectory analysis using ArchR (*60*), constructing a pseudo-time axis by aligning cells based on their culture day. This analysis revealed that *RORC* and other ROR family members were most accessible at earlier time points of in vitro Tr1 differentiation, along with TCR-inducible TFs such as *NFATC2* and NFκB family TFs (fig. S7B). In contrast, TFs *IRF4* and *BATF* were the most accessible later in the Tr1 differentiation process (fig. S7B). Accordingly, the pseudo-time axis started in the cytotoxic Tr1 subcluster C6 and finished in subcluster C9 (Fig. 3I), which was enriched for cells analyzed at day 9 (fig. S6G) and contained activated Tr1 cells expressing cell cycle–related genes such as *G0S2* (fig. S6H and table S7). Both motif accessibility and expression of *IRF4* and *BATF* increased in Tr1 cells along pseudo-time axis, while motif accessibility and gene expression of *MAF* gradually decreased (Fig. 3J) but remained overall high in all Tr1 cells (Fig. 3K). In addition, the expression of *MAF* significantly correlated with the activity of TFs such as *BHLHE40* and *PRDM1* (fig. S7C), which regulate IFN-γ and IL-10, key cytokines produced by Tr1 cells.

Last, we compared the gene expression and TF motif accessibility of Tr1 cells (subclusters C1, C2, C3, C6, and C9) to all other cells in the sc-multiome dataset. Tr1 cells significantly overexpressed genes encoding coinhibitory proteins *LAG3* and *CTLA4*; cytokines *IL10*, *IFNG*, *IL21*, *IL32*, and *VEGFA*; granzymes; chemokine *CCL5*; chemokine receptor *CXCR6*; NADase *CD38*; and TFs *MAF*, *IRF4*, *RBPJ*, *ZEB2*, *TOX2*, *VDR*, *FOXB1*, and *BHLHE40*, among others (Fig. 3L and table S9. Motifs significantly accessible in Tr1 cells included *BATF*, *IRF4*, *MAF*, *VDR*, and *EOMES* (fig. S7D and table S10). In summary, longitudinal sc-multiome analysis of antigen-induced Tr1 cell differentiation confirmed that Tr1 cells have a distinct transcriptional signature and epigenetic program, identifying many of the same TFs as in previous analyses: *MAF*, *BHLHE40*, *ZEB2*, and *RBPJ* (Figs. 1 and 2). In addition, sc-multiome analysis revealed that certain genes and TFs, including *IRF4*, *BATF*, *VDR*, and *TOX2*, are up-regulated in both Tr1 cells and aT_reg_ cells. These shared TFs could govern functions that are common to both antigen-induced Tr1 cells and activated FOXP3-expressing T_reg_ cells or T_reg_ cell–like cells, such as the regulation of *CTLA4*.

### IRF4, BATF, and MAF regulate human antigen-induced Tr1 cells

Next, we investigated the biological significance of the top TFs we identified by transcriptional and epigenetic profiling of Tr1 cells. We started with coregulated TFs *IRF4* and *BATF* (*61*–*63*), which are used by both Tr1 cells and aT_reg_ cells, and *MAF*, the activity of which was highest in Tr1 cells in multiple analyses (Figs. 1 to 3). In addition, we investigated the role of *EOMES* despite its low expression in our in vitro antigen-induced Tr1 cells (fig. S4A) because of its significantly accessible motifs and footprints (Figs. 1, C and D and 3H) and role in Tr1 cells as described by other investigators (*22*, *31*, *46*).

**Fig. 4.**
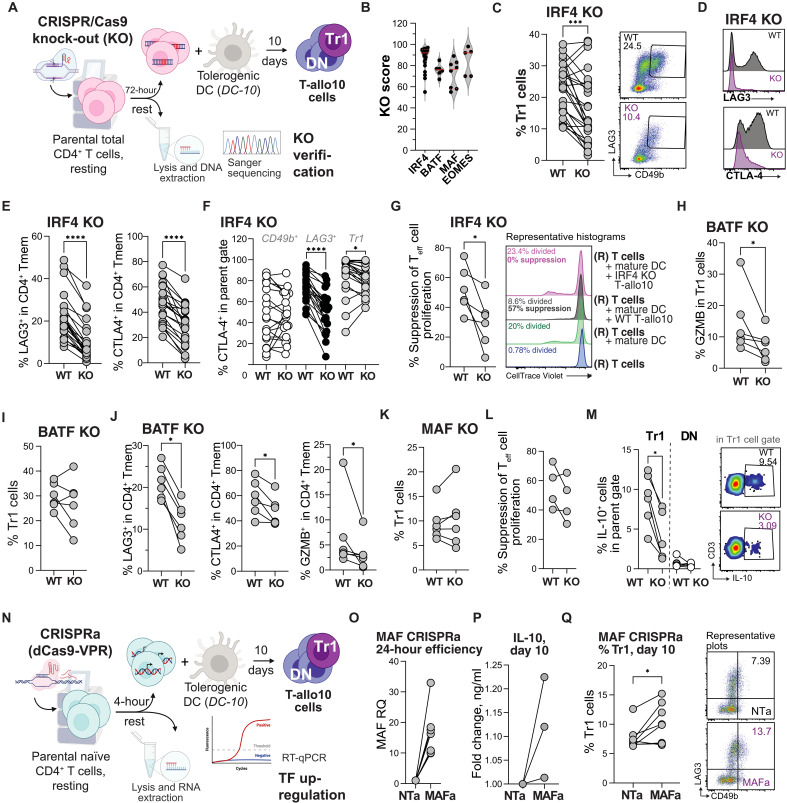
TFs *IRF4*, *BATF*, and *MAF* play nonredundant roles in Tr1 biology. All panels: flow cytometry unless otherwise specified; representative dot plots shown for selected analyses. (**A**) Experimental outline, transcription factor KOs and Tr1 differentiation. (**B**) KO score (percent) 72 hours postediting. Sanger sequencing, ICE (Inference of CRISPR Edits) analysis. Red lines denote median and IQR. *IRF4*, *n* = 25; *BATF*, *n* = 6; *MAF*, *n* = 8; *EOMES*, *n* = 5. (**C**) Tr1 cell frequency within memory (Tmem) T-allo10 cells; WT or *IRF4* KO. *n* = 25. (**D**) Representative histograms, T-allo10 Tmem. (**E**) Frequency of LAG3^+^CD49b^−^ and CTLA-4^+^ cells, WT, or *IRF4* KO T-allo10; *n* = 25. (**F**) CTLA-4 expression in CD49b- or LAG3 single-positive cells and Tr1 cells, T-allo10 Tmem, WT, or *IRF4* KO. *n* = 25. (**G**) Suppression of responder (R) T cell proliferation by WT or *IRF4* KO T-allo10 cells, with representative histograms. *n* = 6. (**H**) Granzyme B (GZMB) expression, WT or *BATF* KO T-allo10 cells; *n* = 6. (**I**) Tr1 frequency, T-allo10 Tmem, WT, or *BATF* KO. *n* = 6. (**J**) Frequency of LAG3^+^CD49b^−^, CTLA-4^+^, and GZMB^+^ T-allo10 Tmem, WT, or *BATF* KO. *n* = 6. (**K**) Tr1 frequency, WT, or *MAF* KO T-allo10 Tmem. *n* = 6. (**L**) Suppression assay (as in G), WT or MAF KO. *n* = 4. (**M**) IL-10–producing Tr1 or CD49b^−^LAG3^−^ (DN) cells, WT or *MAF* KO, within T-allo10 Tmem. *n* = 5. (**N**) Experimental outline, CRISPRa-mediated TF overexpression and Tr1 differentiation. dCas9-VPR = nuclease-inert Cas9 fused to synthetic TFs. (**O**) Relative quantification (RQ) of *MAF*, 24 hours post-CRISPRa. qPCR, *n* = 8. (**P**) IL-10 in T-allo10 supernatants, fold change. Enzyme-linked immunosorbent assay, *n* = 3. (**Q**) Tr1 frequency, nontargeted (NT) and *MAF* CRISPRa, T-allo10 Tmem; *n* = 8. *P < 0.05, ***P < 0.001, and ****P < 0.0001; Wilcoxon test.

Our first approach was to knockout (KO) the target TFs in parental CD4^+^ T cells using CRISPR-Cas9 before Tr1 differentiation in T-allo10 culture (Fig. 4A). To avoid perturbing the epigenome with prestimulation of the TCR, which is used to improve CRISPR efficacy, we knocked out the TFs from unstimulated, ex vivo isolated CD4^+^ T cells with single guide RNAs (sgRNAs) carefully designed to disrupt the universal second exon in *IRF4* and *EOMES* and the first exon in *BATF* and *MAF* genes (fig. S8A). The multiplex sgRNA strategy achieved good KO efficiencies in unstimulated CD4^+^ T cells (80% average; Fig. 4B). After 10 days of in vitro differentiation, T-allo10 cell products generated from KO and control-treated wild-type (WT) parental CD4^+^ T cells were compared for Tr1 cell frequency, phenotype, and function. In some experiments, we measured the TF expression using flow cytometry at the end of T-allo10 culture, showing that the expression of *IRF4*, *MAF*, and *EOMES* was higher in Tr1 than non-Tr1 cells and decreased in Tr1 cells in TF KO condition without changing the expression of other TFs (fig. S8B). Viability and cell quantity, measured using Trypan Blue, were compared between TF KO and control conditions pre- and postelectroporation and after T-allo10 culture. There was no difference in viability in any of the tested conditions. Cell quantity was also not affected, except a small decrease postelectroporation observed in *IRF4* KO condition (*P* = 0.03, Wilcoxon test).

Total T-allo10 cells expanded less after *IRF4* KO than control treatment (median cell count ratios of day 10 to day 0 = 0.82 and 1.36, respectively; *P* = 3 × 10^−5^, Wilcoxon test). This result was driven by a strong and significant decrease in frequency of Tr1 cells, identified by the coexpression of CD49b and LAG3, within T-allo10 cell products (Fig. 4C). The expression of LAG3 and CTLA-4 was significantly decreased in memory CD4^+^ T cells after *IRF4* KO (Fig. 4, D and E). CTLA-4 expression was also significantly decreased in LAG3-single-positive memory CD4^+^ T cells and Tr1 cells (Fig. 4F). *IRF4* KO did not affect the expression of granzyme B (fig. S8C). The few remaining WT CD4^+^ T cells outgrew the *IRF4* KO CD4^+^ T cells in the Tr1 compartment, but not in the non-Tr1 memory or naïve CD4^+^ T cells in *IRF4* KO T-allo10 products (fig. S8D), suggesting that IRF4 provides a competitive growth advantage to in vitro differentiating Tr1 cells. Last, we observed a significant impairment of the suppressive function of *IRF4* KO T-allo10 cells (Fig. 4G), which indicates that IRF4 regulates Tr1 cell differentiation and not just their phenotype.

*BATF* KO significantly impaired the expression of granzyme B in Tr1 cells (Fig. 4H), but did not affect Tr1 cell differentiation (Fig. 4I) and CTLA4 expression in Tr1 cells (fig. S8E). *BATF* KO also impaired the ability of total memory CD4^+^ cells to express LAG3, CTLA-4 and granzyme B (Fig. 4J). *MAF* KO did not affect the Tr1 cell differentiation (Fig. 4K), the expression of LAG3 or CTLA-4 (fig. S8F), or the suppressive function of T-allo10 cells (Fig. 4L). However, in accordance with its described role in regulating IL-10, *MAF* KO significantly impaired the production of IL-10 in Tr1 cells (Fig. 4M). Last, despite its epigenetic activity in Tr1 cells (Fig. 3, B and C), *EOMES* KO did not affect antigen-induced Tr1 cell differentiation (fig. S8, G to J), perhaps due to its expression in only a subset of Tr1 cells (fig. S4A). *EOMES* KO also did not consistently affect Tr1 cell ability to degranulate (fig. S8J).

While IL-10 is not the only mediator of Tr1 suppressive function (*7*), it drives the differentiation of human Tr1 cells. Hence, we asked whether overexpression (OE) of *MAF*, which regulates IL-10 in Tr1 cells (Fig. 4M), increases Tr1 cell abundance after in vitro differentiation. We designed sgRNA guides to target the *MAF* promoter region, and up-regulated *MAF* in naïve CD4^+^ T cells using CRISPR activation (CRISPRa), which induces transient (3 to 5 days) OE of the target gene (Fig. 4N) (*64*, *65*). Viability and cell quantity pre- and postelectroporation and after T-allo10 culture were equivalent between *MAF* activation and nontargeted conditions. We achieved strong up-regulation of *MAF* gene 24 hours after CRISPRa (Fig. 4O) and observed a nonsignificant trend toward higher soluble IL-10 in supernatants of *MAF* OE T-allo10 cells at day 10 (Fig. 4P). Notably, Tr1 cell abundance in *MAF* OE T-allo10 cells was significantly increased compared to controls (Fig. 4Q).

In summary, we show that *IRF4*, *BATF*, and *MAF*, identified by the bioinformatic analyses as top transcriptional regulators of in vitro–induced antigen-specific Tr1 cells, play key roles in regulating Tr1 cell differentiation, phenotype, and functions. These data demonstrate that the transcriptional and epigenetic programs we identified in Tr1 cells through bioinformatic analysis are biologically relevant.

### Tr1 signature identifies Tr1 cells among peripheral blood CD4^+^ T cells of patients treated with Tr1-enriched T-allo10 therapy

Tr1 cells isolated from the peripheral blood of healthy donors are suppressive after in vitro restimulation (*5*), and their transcriptional signature significantly overlaps with in vitro–induced Tr1 cells (fig. S9A). Analyzed ex vivo, circulating Tr1 cells do not express high levels of many TCR-inducible genes such as cytokines, chemokines, and coinhibitory molecules (fig. S9A). The expression of CTLA-4 protein in ex vivo–isolated Tr1 cells, while significantly higher than in non-Tr1 memory CD4^+^ T cells (fig. S9B), is well below the CTLA-4 expression in in vitro–induced Tr1 cells (figs.S4B and S5A). Because of these differences, we investigated if the distinct signature of in vitro–induced Tr1 cells, rather than a limited set of markers, can detect Tr1 cells in vivo*.*

**Fig. 5.**
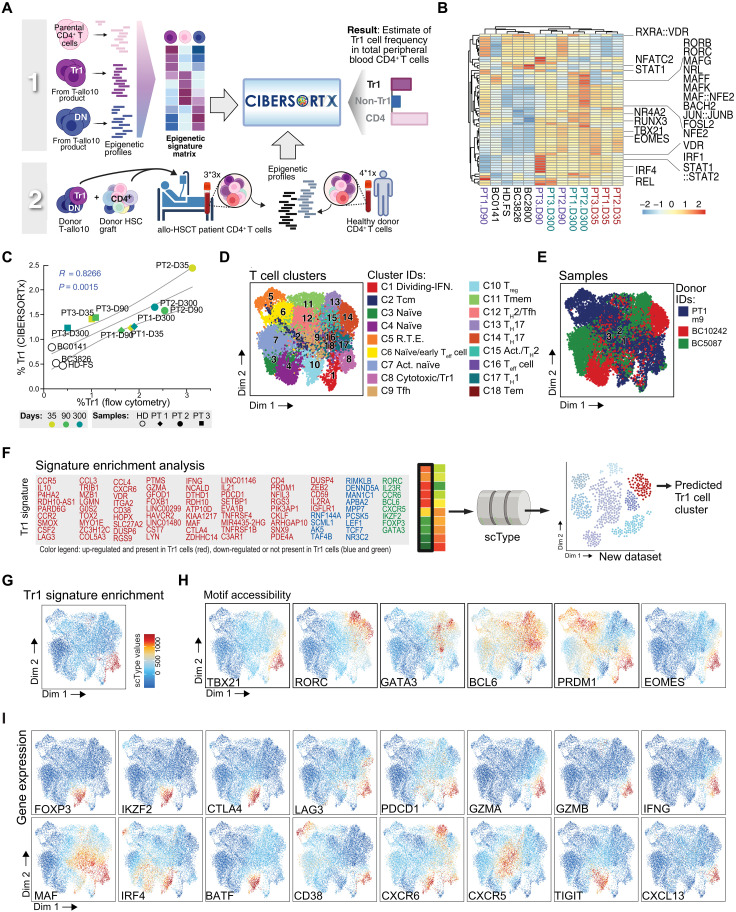
Identification of Tr1 cells in vivo in peripheral blood CD4^+^ T cells. (**A**) Experimental outline, CIBERSORTx-based deconvolution of Tr1 cell abundance from peripheral blood CD4^+^ T cells of patients with allo-HSCT treated with T-allo10 cells. (**B**) Heatmap of TF motif accessibility in CD4^+^ T cells, identified as specific to T-allo10–derived Tr1 cells by CIBERSORTx algorithm. HD and BC = healthy donors; PT = patients 1 to 3; days 35, 90, and 300 indicate days post–T-allo10 infusion and allo-HSCT. (**C**) Frequency of Tr1 cells estimated by digital cytometry (CIBERSORTx; *y* axis) and measured by flow cytometry (*x* axis) in total CD4^+^ T cells; Spearman rank correlation. Line indicates linear regression trendline. (**D**) UMAP embedding with cluster annotations, sc-multiome analysis of peripheral blood CD4^+^ T cells from two healthy donors and one patient treated with T-allo10 infusion and allo-HSCT. (**E**) Distribution of donors. BC = healthy donor, PT = patient; m9 = 9 months post–T-allo10 infusion and allo-HSCT. (**F**) Computational analysis workflow applied for the identification of Tr1 population using ScType and Tr1 signature described in table S12. (**G**) Tr1 score derived from ScType using the Tr1 gene expression signature specific to in vitro antigen-induced Tr1 cells in CD4^+^ T cells of healthy donors and a patient analyzed by sc-multiome. (**H**) Motif accessibility of TFs that act as key regulators of T-helper cell subsets, sc-multiome analysis. (**I**) Gene expression of selected genes characteristic for Tr1 cells, cytotoxic T cells, and FOXP3^+^ T_reg_ cells, sc-multiome analysis.

Toward this goal, we first applied CIBERSORTx, a digital cytometry method that estimates abundances of target cell types in a heterogenous bulk population using high-throughput data (*66*). To generate the samples for CIBERSORTx, we purified live total CD4^+^ T cells from cryopreserved peripheral blood mononuclear cells (PBMCs) of three patients at days 35, 90, and 300 after T-allo10 cell infusion and allo-HSCT (*7*, *67*) (ClinicalTrials.gov ID: NCT03198234) and analyzed them by ATAC-seq (Fig. 5A). We also analyzed healthy donor CD4^+^ T cells, which contained significantly fewer CD49b^+^LAG3^+^ Tr1 cells than in T-allo10 recipients (fig. S9, C and D) (*7*), to test the threshold of the method. To construct a CIBERSORTx TF signature matrix (Fig. 5A), we used the interactive CIBERSORTx user interface (*66*) and the TF motif data from purified parental CD4^+^ T cells, Tr1, and non-Tr1 cells from the T-allo10 cell products (table S11). These samples were chosen to parallel CD4^+^ T cells present in patient’s peripheral blood, which are comprised from parental CD4^+^ T cells from the allo-HSCT graft and T-allo10 cells. Next, we investigated the relative accessibility of TF motifs, identified by the CIBERSORTx algorithm as characteristic for Tr1 cells (table S11), within the bulk CD4^+^ T cells. All but one patient sample clustered apart from healthy donor samples (Fig. 5B), and the accessibility of some TFs varied by group (healthy versus patients) or time point post-HSCT. For example, the relative accessibility of *EOMES* and *IRF4* motifs was elevated in patients at all time points compared to healthy donors, the accessibility of *NFATC2* and *VDR* was similar across groups and time points, and the accessibility of *MAF::NFE2* and *JUN::JUNB* motifs was higher in patients and elevated further at day 300 (fig. S9E).

Using the TF signature matrix and TF motif accessibility, CIBERSORTx algorithm determined Tr1 cell frequencies in bulk CD4^+^ T cell samples, which we compared to Tr1 cell frequencies detected by flow cytometry. These data revealed that the frequencies of Tr1 cells measured by CIBERSORTx and flow cytometry were similar (fig. S9F) and that the frequency of Tr1 cells identified by digital cytometry significantly correlated with the frequency of Tr1 cells identified by flow cytometry (Fig. 5C). These results confirm our previous TCR clonotype tracking indicating that Tr1 cells persist long-term in vivo after adoptive transfer (*7*) and suggest that the Tr1 TF motif signature identified in in vitro antigen-induced Tr1 cells can be leveraged to detect Tr1 cells in vivo.

Next, we investigated whether the Tr1 gene expression signature could be used to identify Tr1 cells in vivo, across different conditions and datasets where the expression of individual Tr1 markers may vary. To define the Tr1 gene signature, we selected genes that were significantly up-regulated or down-regulated in Tr1 cells compared to all other cells, both at the single-cell level (Fig. 3L) and in our published bulk RNA-seq data (*7*). This analysis identified 68 common up-regulated and 13 common down-regulated genes (table S12). To enhance the distinction between Tr1 cells and other CD4^+^ T cell subsets with overlapping transcriptional profiles, we expanded the down-regulated gene list by including canonical markers of other CD4^+^ T cell lineages: *FOXP3* and *IKZF2* (FOXP3^+^ T_reg_ cell markers), *RORC*, *IL23R*, and *CCR6* (T_H_17 markers); *BCL6* and *CXCR5* (T_FH_ cell markers); and *GATA3* (a T_H_2 marker) (table S12). This Tr1 gene signature was used with ScType (*68*) algorithm to automate cell annotation and distinguish between Tr1 and non-Tr1 cells.

We generated a sc-multiome dataset of CD4^+^ T cells purified from cryopreserved PBMC of three healthy donors and one leukemia patient treated with T-allo10 cells and allo-HSCT at 9 months posttreatment. One of the healthy donors had very high LAG3 single-positive population detected by flow cytometry and was removed as a statistical outlier (fig. S10). The remaining two healthy donors had ~1% of Tr1 cells detected by flow cytometry, while the patient had 6.42% of Tr1 cells (fig. S10A). Patient and healthy donor sc-multiome samples were integrated, and 14,405 cells clustered, revealing CD4^+^ T cell clusters that were annotated based on the representative genes (Fig. 5, D and E, and table S13). The ScType analysis identified cluster C8 as the most likely Tr1 cell cluster (Fig. 5, F and G). Cells from the T-allo10-treated patient sample comprised 83.4% of cluster C8 (Fig. 5E and fig. S11A).

To confirm the ScType annotation of C8 as the Tr1 cell cluster, we examined the expression of TFs and genes archetypal for Tr1 cells and other T_H_ cell subsets. TBX21 motif was highly accessible in most of the cells in cluster C8 and also accessible in the T_H_1 cluster C17 (Fig. 5H) (*69*). TF motifs characteristic for T_H_2, T_H_17, and T_FH_ cell cells (*GATA3*, *RORC*, and *BCL6*, respectively) were inaccessible in cluster C8 (Fig. 5H). Cells in cluster C8 had accessible TF motifs for *PRDM1* and *EOMES*, aligned with the Tr1 phenotype. FOXP3^+^ T_reg_ cells were identified in cluster C10, which had high gene expression of *FOXP3*, Helios (encoded by *IKZF2*), and *CTLA4* (Fig. 5I). *CTLA4* had lower expression in cluster C8, which is consistent with resting Tr1 cells found in peripheral blood (fig. S9, A and B). Cells in cluster C8 expressed *CD38*, *LAG3*, *PDCD1*, *GZMA*, *GZMB*, and *IFNG*, the latter of which was also up-regulated in T_H_1 cluster C17 (Fig. 5I). TFs *IRF4* and *BATF* had the highest expression in FOXP3^+^ T_reg_ cells, while the expression of *MAF* was highest in Tr1-predicted cluster C8. *MAF* was also expressed in clusters of effector T cells (C16, C17, and C18) and in cluster C9. C9 had elevated *CXCR5* and intermediate levels of *TIGIT* (Fig. 5I), which is consistent with a T_FH_ cell phenotype. A chemokine sometimes associated with T_FH_ cell and peripheral helper T (Tph) cells, *CXCL13* (*70*), was also expressed in cluster C8 (Fig. 5I). Last, chemokine receptor *CXCR6*, which we identified as epigenetically active and highly expressed in in vitro–induced Tr1 cells (Figs. 1, G to I, 2D, and 3L), was expressed not only in cluster C8 but also in T_H_17 and T_reg_ cell clusters (Fig. 5I).

The expression of archetypal Tr1 genes in cluster C8 was in alignment with ScType enrichment result, but it is possible that C8 contains a mixture of Tr1 cells and cytotoxic or T_H_1 cells. For example, cluster C8 contained cells that expressed high levels of Tr1-associated genes *LAG3*, *CXCR6*, and *CD38* and cells that had only background expression level of these markers. Hence, we annotated cluster C8 as Cytotoxic/Tr1. In summary, these data show that Tr1 cell signatures, identified in alloantigen-induced T-allo10–derived Tr1 cells in vitro, can be used to estimate the quantity and identity of Tr1 cells among peripheral blood CD4^+^ T cells of patients treated with T-allo10 cell products.

### Tr1-specific transcriptional signature identifies Tr1 cells across human solid tumors

Tr1 cells are more likely to encounter antigens, which will up-regulate genes required for suppressive activity such as *CTLA4*, in the tissues rather than peripheral blood. However, CD49b and LAG3 may not be reliable Tr1 markers in human tissues (*22*, *26*). To investigate if our Tr1 signature and ScType can identify Tr1 cells in tissues, we focused on solid tumors, where recent studies suggested that Tr1 cells could play a detrimental role (*22*, *23*). We reanalyzed CD4^+^ T cells from public single-cell datasets of tumors with moderate or high tumor mutational burden (TMB). We selected these tumors because TMB correlates, to an extent, with tumor antigen load (*71*, *72*), and Tr1 cells are antigen-inducible.

**Fig. 6.**
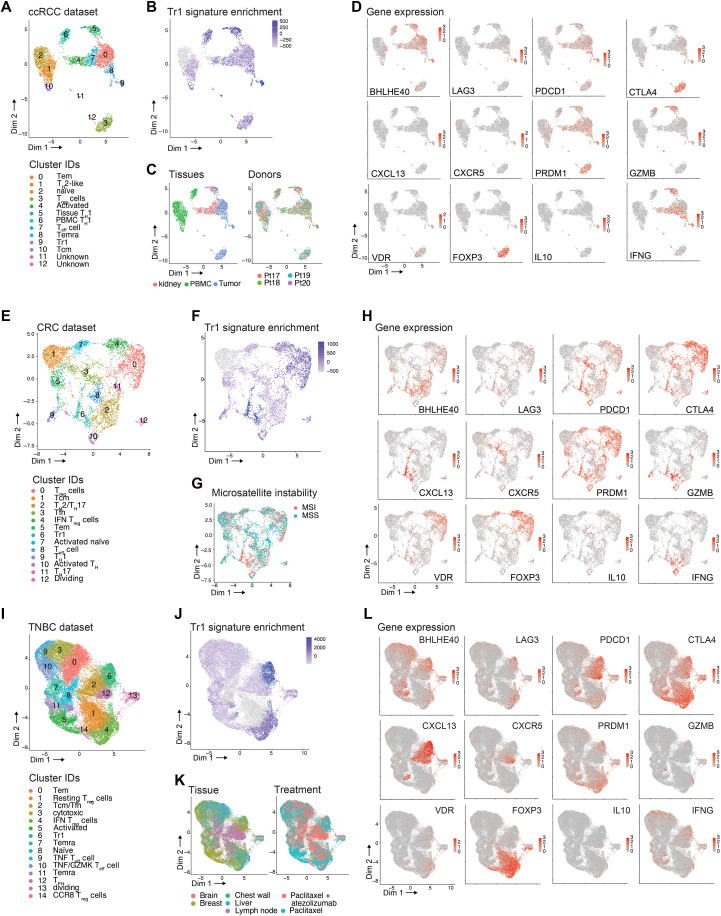
Identification of Tr1 cells in vivo among solid tumor-resident human CD4^+^ T cells. (**A**, **E** and **I**) UMAP clusters of re-analyzed public datasets of integrated ccRCC, CRC, and TNBC datasets, respectively, after computational isolation of CD4^+^ T cells, with knowledge-based annotation of CD4^+^ T cell populations. (**B**, **F**, and **J**) Enrichment of the Tr1-specific gene expression signature in indicated datasets using ScType. (**D**, **H**, and **L**) UMAP embedding displaying gene expression levels of canonical Tr1 markers. (**C**) Tissue and donor distribution of CD4^+^ T cells in ccRCC dataset. (**G**) Distribution of cells from CRC samples that have mismatch repair deficiency, also known as microsatellite instability (MSI) or are microsatellite stable (MSS). (**K**) Tissue and treatment distribution of samples from the TNBC dataset.

First, we reanalyzed 4161 CD4^+^ T cells from the peripheral blood, tumor tissue, and adjacent normal tissue of four patients with stage I clear-cell renal cell carcinoma (ccRCC) (*73*) while filtering out CD8^+^ T cells, B cells, natural killer (NK) cells, and myeloid cells. Samples were integrated, identifying 13 clusters that we annotated using T_H_ subset characteristic genes (Fig. 6A and table S14). ScType identified cluster C9 as most likely to be Tr1 cells (Fig. 6B), which was composed of tumor-derived CD4^+^ T cells from all four patients (Fig. 6C and fig. S11B). Cells in cluster C9 expressed *BHLHE40*, *LAG3*, *PDCD1*, *CTLA4*, *PRDM1*, *GZMB*, *IFNG*, *IL10* (Fig. 6D), *HAVCR2*, *CCL5*, and *TIGIT* (fig. S11C), but not T_reg_ cell gene *FOXP3* or T_FH_ cell gene *CXCR5* (Fig. 6D). In this dataset, the expression of *CXCL13* was sparse (Fig. 6D), but the transcriptional profile of cells in the predicted Tr1 cluster was overall in accordance with the known Tr1 profile.

The same study included a scATAC-seq dataset of eight patients with stage I ccRCC (*73*), which was used to test ScType functionality with chromatin accessibility data. We integrated the samples and identified 12 CD4^+^ T cell clusters (fig. S12, A to D, and table S14), which were separated according to the tissue type (fig. S12, E and F). Using ScType and peaks annotated to nearest genes, cluster C6, composed of tumor CD4^+^ T cells, was identified as the most likely Tr1 cell cluster (fig. S12G). Cluster C6 had accessible motifs for *TBX21*, *MAF*, *BATF*, and *IRF4*, but closed motifs characteristic for T_H_2, T_H_17 cells, and T_FH_ cell cells (*FOXP3, GATA3, RORC*, and *BCL6*, respectively) and closed *FOXP3* gene (fig. S12H). *CTLA4* accessibility was lower in the tumor Tr1 cluster C6 than in blood and tumor FOXP3^+^ T_reg_ cells (fig. S12I), and *IL10* accessibility was higher but did not reach statistical significance (fig. S12I). Perforin (*PRF1*) accessibility was expectedly high in T_H_1 cluster C3 and intermediate in cluster C6 (fig. S12I). Cluster C5 also had moderately high Tr1 cell signature enrichment by ScType (fig. S12G), so we compared clusters C5 and C6 (table S15). The accessibility of *IL17RE* was highest in cluster C5 (fig. S12I), likely indicating T_H_17-like cells. Cluster C6 had significantly more accessible TFs and genes we previously identified in Tr1 cells, including *GZMK* (table S8), *CEBPD* (Fig. 2L), *CD274* (encoding PD-L1) (*7*), *CXCR6* (Figs. 1 to 3), and *CCL3* (Fig. 1H and table S12). *CXCL13* was also more accessible in cluster C6 (fig. S12I) in the absence of T_FH_ cell TF *BCL6* or the Tph TF *SOX4* (fig. S12I) (*70*). These data show that Tr1 transcriptional signature could identify clusters of cells with Tr1 features in both scRNA- and ATAC-seq data, albeit with less precision in ATAC-seq data.

Next, we reanalyzed scRNA-seq data of CD4^+^ T cells from the blood, normal colon, and tumors of 12 patients with CRC (*74*), 4 of whom had microsatellite instable (MSI) tumors associated with high TMB (*75*). Samples were integrated, and 4902 CD4^+^ T cells formed 12 clusters (Fig. 6E and table S14). ScType identified cluster C6 as the likely Tr1 cell cluster (Fig. 6F). Cluster C6 derived predominately from patients with MSI CRC (Fig. 6G). Cells in cluster C6 expressed *BHLHE40*, *LAG3*, *PDCD1*, *CTLA4*, *PRDM1*, *VDR*, *GZMB*, *IFNG* (Fig. 6H), *CCL3*, *HAVCR2*, *TIGIT*, *RBPJ*, and *MAF* (fig. S13A), but not the T_reg_ cell gene *FOXP3* or T_FH_ cell gene *CXCR5* (Fig. 6H). The expression of *CXCL13* was observed in Tr1 cluster C6 and in the T_FH_ cell cluster C3 (Fig. 6H). The cluster of *BHLHE40*^+^*CXCL13*^+^ CD4^+^ T cells was also identified in the original study by Zhang *et al.* (*74*), where they were annotated as T_H_1-like cells that were clonally expanded and proliferative, and found mostly within tumor tissue (fig. S13B) of patients with the MSI, but not MSS tumors.

We also reanalyzed scRNA-seq data of tissue CD4^+^ T cells of 22 patients with triple-negative breast cancer (TNBC) (*76*), which has higher TMB (*72*) than other subtypes of breast cancer, and can respond to checkpoint inhibitors. Samples were integrated, and 31,523 CD4^+^ T cells were separated into 15 clusters (Fig. 6I and table S14). ScType identified cluster C6 as the likely Tr1 cell cluster (Fig. 6J), which was composed from cells isolated from the breast cancer and the chest wall, and present both in chemotherapy (paclitaxel)– and combination therapy (paclitaxel + atezolizumab, anti–PD-L1)–treated patients (Fig. 6K). Cells in cluster C6 expressed *BHLHE40*, *LAG3*, *PDCD1*, *CTLA4*, *PRDM1*, *IFNG* (Fig. 6L), *CCL5*, *CD38*, *TIGIT*, *RBPJ*, *MAF* (fig. S13C), and low *GZMB* and TF *VDR* (Fig. 6L), but not the T_reg_ cell gene *FOXP3* or T_FH_ cell gene *CXCR5* (Fig. 6L). *CXCL13* chemokine was expressed in Tr1 cluster C6 and in the T_FH_ cell clusters C2 and C12 (Fig. 6L). In the original study, Zhang *et al.* (*72*) observed that total *CXCL13*^+^ *CD4*^+^ T cells decrease in patients that respond to chemotherapy but remain the same or expand in patients that respond to combination therapy. Similarly, total FOXP3^+^ T_reg_ cells decreased in responders to chemotherapy but did not significantly change after combination therapy (*72*). Our reanalysis separated *CXCL13*^+^ *CD4*^+^ T cells into three subclusters, two T_FH_ cell and one Tr1 cluster; the Tr1 fraction of *CXCL13*^+^ CD4^+^ T cells appeared to decrease in responders after both chemotherapy and combination therapy (fig. S14, A and B). As well, our reanalysis separated *FOXP3*^+^ T_reg_ cells into three populations, two of which exhibited signs of activation, including high expression of *LAYN* [clusters C4 (IFN T_reg_ cells) and C14 (CCR8^HIGH^ T_reg_ cells); Fig. 6E and table S14]. Same as Tr1 cells, both clusters of activated FOXP3^+^ T_reg_ cells decreased in responders to chemotherapy and combination therapy (fig. S14, C to E). In contrast, T_eff _ cells increased in patients that responded to therapy (*72*). Thus, the dynamics of Tr1-like cells in patients with TNBC paralleled the dynamics of activated FOXP3^+^ T_reg_ cells, suggesting a similar biological role.

Last, we reanalyzed matched TCR-seq data from those studies. In each tumor, Tr1 clusters were among the top four clusters with most abundant large and medium clones (fig. S15), suggesting that Tr1 cells clonally expand in the tumor microenvironment in response to antigen.

### Tr1-specific transcriptional signature identifies murine Tr1 cells in a solid tumor model

**Fig. 7.**
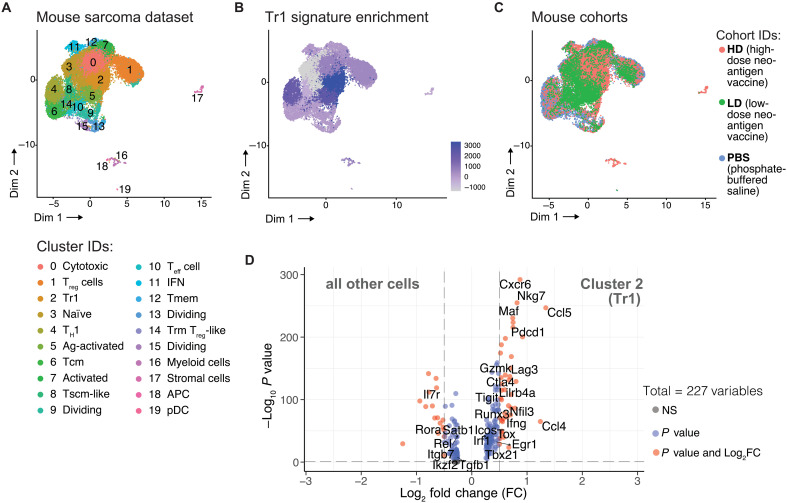
Identification of bona fide murine solid tumor Tr1 cells using human Tr1 transcriptional signature. (**A**) Unsupervised clustering of scRNA-seq data from mouse sarcoma dataset from (*23*) (*n* = 26,980 cells). Twenty distinct clusters were identified and annotated based on the expression of canonical markers. (**B**) Enrichment of the human Tr1 transcriptional signature across the identified murine clusters, visualized on a blue color scale (low to high enrichment). (**C**) Distribution of cells across three experimental cohorts: high-dose neoantigen vaccine (HD), low-dose neoantigen vaccine (LD), and PBS control. (**D**) Differential expression analysis of the predicted Tr1 cluster [identified in (B)] compared to the remaining cell populations. pDC, plasmacytoid dendritic cells; APC, antigen-presenting cell.

To corroborate the Tr1 signature enrichment analysis results, we turned to the published murine sarcoma model (*23*), where Tr1 cells were induced in response to high-dose but not low-dose neoantigen vaccine. These neoantigen-specific Tr1 cells suppressed tumor rejection in vivo after adoptive transfer of T_eff _ cells or anti-PD1 treatment, which did not occur if Tr1 cells were depleted. We reanalyzed the published scRNA-seq data from this study (*23*), identifying 20 clusters that were annotated using canonical markers (Fig. 7A). Human Tr1 signature enrichment was strongest in cluster 2 (Fig. 7B), which predominantly contained cells from mice treated with high-dose neoantigen vaccine (82.7%; Fig. 7C). The differential gene expression analysis between cluster 2 and all other clusters identified archetypal Tr1 genes significantly up-regulated in cluster 2, such as *Lag3*, *Ccl5*, *Cxcr6*, *Maf*, *Pdcd1*, *Ctla4*, *Ifng*, *Gzmk*, and *Nfil3* (Fig. 7D), which is aligned with the murine Tr1 signature from the original study. Also, cells in cluster 2 also expressed *Lilrb4*, a murine-only Tr1 cell marker (*23*).

In summary, we show that human Tr1 transcriptional signature, derived from in vitro antigen-induced Tr1 cells, identifies clusters of cells exhibiting Tr1 features and signs of clonal expansion in human solid tumors with high or moderately high TMB (and thus likely higher neoantigen load), but not in tumors with low TMB. Notably, human Tr1 signature enrichment analysis accurately identified a cluster of tumor antigen-specific, bona fide murine Tr1 cells, which were induced in vivo in response to neoantigens.

## DISCUSSION

Despite increasing interest in Tr1 biology in various antigen-driven inflammatory conditions (*14*–*22*, *77*) and promising early results from ongoing clinical trials with Tr1-enriched T-allo10 cell therapy in allo-HSCT (*7*, *67*, *78*), the epigenetic landscape of human Tr1 cells and their transcriptional regulators has not been systematically investigated. We show that human in vitro–induced, antigen-specific Tr1 cells have a unique transcriptome, oligoclonal TCR repertoire, and a distinct signature of open chromatin, TF motifs, and TF footprints in comparison to the parental CD4^+^ T cells and other CD4^+^ cell populations. Functional genomics confirmed that TFs identified in these analyses as most likely Tr1 regulators—*IRF4*, *BATF*, and *MAF*—play important and largely nonredundant roles in Tr1 cell differentiation, phenotype, and functions. The Tr1 cell transcriptional signature identified in antigen-induced Tr1 cells in vitro can also reveal Tr1 cells in vivo, in scRNA-seq data of T-allo10 recipients and patients and with solid tumors, and identify bona fide murine Tr1 cells induced in response to neoantigens in vivo.

There is a current lack of consensus on the identity of Tr1 cell–regulating TFs. In mice, where Tr1 cells are induced in the presence of IL-27, key transcriptional regulators of Tr1 cells depend on the differentiation model. A polyclonal model revealed *Irf1*, *Batf*, *Maf*, and *Ahr* as murine Tr1 lineage-defining TFs (*28*–*30*), an antigen-specific model pointed to *Eomes*, *Tbx21*, and *Prdm1* (*31*, *79*), while a peptide-nanoparticle model pointed to *Bcl6*, *Irf4*, and *Prdm1* (*3*). In humans, Tr1 cell induction is IL-10–driven, and mechanistic studies to validate the functional role of lineage-defining Tr1 TFs are few. In one study, lentiviral vector-mediated OE of *EOMES* in CD4^+^ T cells induced polyclonal Tr1 cells (*46*), suggesting its role in Tr1 differentiation. Another study used short hairpin RNA and small-molecule inhibitors to show that *IRF4* cooperates with *AHR* to regulate IL-10 production and the suppressive function of activin-A–induced polyclonal Tr1 cells (*80*). We investigated the lineage-defining TFs in human antigen-specific Tr1 cells, which are induced in vitro in response to antigens presented by allogeneic tolerogenic dendritic cells (*7*, *16*, *81*). Our epigenome and transcriptome analysis showed that human antigen-induced Tr1 cells used *IRF4*, *BATF*, and *MAF* as well as up-regulated *TBX21* and *PRDM1*, but not *BCL6* or *AHR* as suggested in other studies (*3*, *80*). As in murine studies, differences in the identified transcriptional regulators of human Tr1 cells could be due to the different experimental models.

We demonstrate that *IRF4*, *BATF*, and *MAF* have high epigenetic activity in human Tr1 cells. These results are aligned with epigenetic priming data from tolerized, Tr1-like murine CD4^+^ T cells, which showed increased occupancy of *Irf4*, *Batf*, and *Maf* at tolerance-associated loci, such as *Il10* (*82*). Our functional genomics experiments confirmed that *IRF4*, *BATF*, and *MAF* regulate key phenotypic traits and functions of antigen-induced human Tr1 cells. We also show that *IRF4* and *BATF*, which often cooperate (*62*), are needed for induction of CTLA-4 and LAG3 in memory CD4^+^ T cells and are up-regulated in activated FOXP3^+^ T_reg_ cells. Thus, IRF4 and BATF may regulate CTLA-4 and LAG3 in other T cell subsets besides Tr1 cells (*83*). Moreover, IRF4 and BATF play a role in T_H_17 differentiation (*84*), and our trajectory analysis suggests that T_H_17 lineage TF *RORC* is active in early cytotoxic Tr1 cells. It is possible that the activation of *IRF4* and *BATF* during Tr1 cell differentiation does not lead to T_H_17 cells due to concomitant activation of lncRNA *NEAT1* and TFs *EOMES* and *TBX21*, which repress the T_H_17 phenotype (*31*, *54*, *59*, *85*). IRF4 also regulates the differentiation of T_FH_ cells (*86*) and the production of their hallmark cytokine IL-21 (*87*). In our hands, antigen-induced Tr1 cells express IL-21 and genes associated with T_FH_ cells such as *ICOS*, but not the T_FH_ cell chemokine receptor *CXCR5* or T_FH_ cell master regulator TF *BCL6* (*88*, *89*). This may be due to the high levels of *PRDM1* in Tr1 cells, which can repress *BCL6* (*88*).

We identified *MAF* as another key transcriptional regulator of antigen-induced human Tr1 cells. *MAF* is a universal regulator of IL-10 production in CD4^+^ T cell subsets (*90*). In mice, *Maf* cooperates with *Ahr* and *Prdm1* to achieve optimal transcription of IL-10 (*91*), and with *Prdm1* to up-regulate the expression of a coinhibitory receptor module that includes *LAG3* (*92*). While we show that *MAF* regulates IL-10 production in human Tr1 cells, *MAF* KO or OE did not change the expression of LAG3. This may be because *PRDM1*, which is highly expressed in Tr1 cells, compensates for the lack of *MAF*. Furthermore, it is conceivable that *MAF* plays a role in human Tr1 differentiation that is distinct from its effect on IL-10 production. We show that even transient OE of *MAF* increased Tr1 cell yields after 10-day in vitro differentiation. Unlike *IRF4* and *BATF*, we did not find *MAF* significantly up-regulated in either resting or activated T_reg_ cells.

Tr1 TF motif signature-based digital cytometry estimated Tr1 cell proportions in a heterogenous bulk population of total peripheral blood CD4^+^ T cells, isolated longitudinally from patients treated with Tr1-enriched T-allo10 cell therapy (*67*). This epigenetic-based tracking confirms our previous TCR clonotype-based tracking in the same patient population up to 300 days posttreatment, which suggested that infused Tr1 cells persist long term in patients (*7*). We also showed that Tr1 cell transcriptional signature and ScType platform can identify cluster of Tr1 cells in scRNA-seq data peripheral blood CD4^+^ T cells of a patient 9 months after T-allo10 cell therapy and allo-HSCT.

The Tr1 transcriptional signature revealed clusters of cells with Tr1 phenotype among tissue-resident CD4^+^ T cells, where the coexpression of CD49b and LAG3 is less reliable (*22*, *26*). Specifically, we show that Tr1 signature identifies cells with Tr1-like features in single-cell datasets of CD4^+^ T cells from human solid tumors. Besides the public datasets we reanalyzed, cells with Tr1 features were described in other human cancer datasets. In MARS-seq dataset of melanoma, a CD4^+^ T cell cluster was annotated as “dysfunctional CD4 T cell” based on expression of coinhibitory genes such as *LAG3*, *CTLA4*, and *PDCD1*, but the same cluster also expressed *MAF*, *CXCL13*, *CCL3*, *IFNG*, *PRDM1*, *CXCR6*, *CCR5*, *HAVCR2*, and *IL10*, which are genes highly indicative for Tr1 cells (*93*). A single-cell study of tumor-infiltrating T cells from 21 human tumors in more than 300 patients described a cluster of “T_FH_ cell/T_H_1” CD4^+^ T cells. These T_FH_ cell/T_H_1 cells, unlike the bona-fide T_FH_ cell cluster, did not express archetypal T_FH_ cell genes *BCL6* and *CXCR5* but up-regulated *CTLA4*, *LAG3*, *HAVCR2*, *IFNG*, *GZMB*, and *CCL3* and had intermediate level of T_H_1 TF *TBX21*, which is again highly suggestive of Tr1 cells (*94*). The T_FH_ cell/T_H_1 cluster was tumor-enriched, clonally expanded, and strongly correlated with the TMB, which is aligned with the antigen-inducible Tr1 cell development.

Critically, the human Tr1 signature we present here can identify bona fide Tr1 cells induced in vivo, which we demonstrate in the reanalysis of scRNA-seq data from a murine sarcoma model (*23*). In that model, neoantigen-specific cytotoxic Tr1 cells mediate resistance to anti–PD-1 therapy and adoptive T cell transfer (*23*). This study, together with our data, suggests that Tr1 cells should be considered in evaluating response to treatment in human solid tumors and in design of therapeutic strategies that decrease T cells with suppressive function in vivo. The depletion of FOXP3^+^ T_reg_ cells with anti-CCR8 antibody (*24*) to improve outcomes of patients with solid tumors is already being investigated in several clinical trials (ClinicalTrial.gov IDs NCT05635643, NCT06387628, NCT05518045, NCT05537740, NCT05007782, NCT05101070, NCT06131398, and NCT05935098). If immune suppression in some of these indications is mediated by Tr1 cells in addition to FOXP3^+^ T_reg_ cells, then T_reg_ cell depletion alone may not be effective. An antibody against LILRB4 depleted murine cytotoxic Tr1 cells and restored antitumor immunity in the above-mentioned solid tumor model (*23*), but this gene is not expressed in human T cells [(*95*); Human Protein Atlas, www.proteinatlas.org] or human Tr1 cells (our data). In the same murine model, Tr1 cells suppressed antitumor responses by killing dendritic cell that activated T_eff _ cells (*23*). We also showed, here and previously (*8*, *10*), that human Tr1 cells are cytotoxic and kill myeloid cells. Together, the ability to identify human Tr1 cells in tumors, which our data will facilitate, will enable investigating the role intratumoral Tr1 cells play in cancer patient outcomes.

Our study has some limitations. We observed that batch correction in scATAC-seq datasets was less effective than in scRNA-seq, and some clusters were sample-driven, possibly resulting from ATAC-seq intrinsic technical bias during library preparation (*96*). This could affect data interpretation. In addition, IRF4 regulates the balance between glycolysis and oxidative phosphorylation in T cells (*83*), and its KO could impair Tr1 cell differentiation due to a metabolic impairment that affects their clonal expansion. However, we did not observe strong *IRF4* activity in control T_eff _ cells, which also clonally expand in response to alloantigens (*7*). Thus, *IRF4* also likely regulates metabolism-independent facets of Tr1 cell biology, such as CTLA-4 and LAG3 expression. Furthermore, the epigenetic and transcriptional signature of Tr1 cells has been derived from in vitro–induced antigen-specific Tr1 cells, which allowed us to validate the top identified TFs via functional genomics. While we used the described Tr1 transcriptional signature to identify cells with Tr1 phenotype in vivo, we have tested it in a limited set of conditions. Because Tr1 cells share features with T_H_1, T_EMRA_, T_FH_ cell, and Tph cells, removing non-CD4^+^ T cells and verifying predicted Tr1 cell clusters by careful annotation is warranted. Tr1 cluster prediction remains to be confirmed in humans by validation of Tr1 cell function ex vivo*.* This study sets a foundation for this approach by defining the Tr1 signature and transcriptional profile of human tumor-resident Tr1 cells, facilitating identification of markers that can be used for their purification.

In summary, we uncover the epigenetic landscape of human antigen-specific Tr1 cells, their key transcriptional regulators *IRF4*, *BATF*, and *MAF* and their transcriptional signature. This signature can detect cells with Tr1 features in vivo in human peripheral blood and among tumor-resident CD4^+^ T cells in humans and mice. Thus, the data provided herein illuminate two key challenges in Tr1 cell biology: their transcriptional regulation and their identification. These data can be used to develop Tr1 cell–based therapies using TF engineering, devise strategies to inhibit Tr1 cell differentiation in vivo using targeted TF degradation approaches, and serve as a basis for mechanistic studies of Tr1 cell biology. Together, our study will advance our understanding of the role Tr1 cells play in health and disease.

## MATERIALS AND METHODS

### Primary cells

Human PBMCs were sourced from buffy coats of deidentified donors without prior knowledge of their demographic information (Stanford Blood Center, Palo Alto, CA, USA). Demographics were obtained for donors used for scRNA-seq experiments (table S16). PBMC was isolated using Ficoll-Paque Plus (GE Healthcare) density gradient. CD4^+^ T cells were isolated from PBMC using Human CD4^+^ T Cell Isolation Kits (Miltenyi Biotec Inc., Bergisch Gladbach, Germany). Naïve CD4^+^ T cells were isolated using the Human Naïve CD4^+^ T Cell Isolation Kit II (Miltenyi Biotec). CD14^+^ monocytes were isolated using Human CD14 Microbeads (Miltenyi Biotec). Cell purity was verified by flow cytometry. CD4^+^ T cells were cultured in a T cell medium of X-VIVO-15 (Lonza, Basel, Switzerland) with 5% Human AB Serum (MilliporeSigma, Burlington, MA, USA). PBMC was isolated from patients, and allo-HSCT donors enrolled in the T-allo10 clinical trial (ClinicalTrials.gov Identifier: NCT03198234) were collected in accordance with the Administrative Panels on Human Subjects in Medical Research, Stanford University T-allo10 eProtocol #38734, after an informed consent. Clinical study design and patient inclusion and exclusion criteria can be found at ClinicalTrials.gov. Demographics, treatment, and clinical data of the patients were reported previously (*67*).

### Flow cytometry

Purified primary cells, tolerogenic dendritic cells (DC-10), mature (mat) DC, T-allo10 cells, and cells used for T cell degranulation and suppression assays were stained to assess purity, phenotype, and/or proliferation via flow cytometry. Cells were first stained for viability for 15 min at room temperature (RT) using 100 μl of a 1:1000 (Live/Dead InfraRed) or 1:500 (Live/Dead Aqua and Live/Dead Violet) fixable viability dye in phosphate-buffered saline (PBS). Cells were then washed and incubated for 5 min at RT with 5 μl of Fc receptor blocking reagent (Miltenyi) in 50 μl of staining buffer composed of PBS with 0.02% sodium azide and 2% fetal bovine serum and then incubated for 15 min at RT using an extracellular antibody cocktail with staining buffer added to 100 μl. Cells were then washed and acquired on a flow cytometer or fixed and stained intracellularly. For cytoplasmic antigens, cells were fixed with BD Cytofix or Cytofix/Cytoperm Buffer (BD Biosciences, San Jose, CA, USA) at +4°C (minimum 20 min up to overnight), washed with saponin-based BD Permeabilization Buffer, and then stained with intracellular antibody cocktail diluted in 50 μl of BD Permeabilization Buffer for 30 min at +4°C. After intracellular staining, cells were washed with Permeabilization Buffer and resuspended in Staining Buffer before acquisition. For intranuclear antigens (e.g., TFs), cells were fixed, permeabilized, and stained using Pharmingen Transcription Factor Buffer (BD Biosciences) or eBioscience FOXP3/Transcription Factor Staining Buffer Set (Invitrogen) following the manufacturer’s instructions. Samples were acquired on a BD FACSymphony A5 or a Beckman-Coulter CytoFLEX. Flow cytometry data were analyzed using FlowJo v10.8 (BD). In all experiments, events were gated on live single cells. Antibodies for each panel are listed in table S17.

### In vitro Tr1 cell differentiation

The T-allo10 protocol from Chen P and Cepika AM *et al.* (*7*) was adapted to accommodate gene expression modulation with CRISPR. Briefly, DC-10 and matDC were differentiated from CD14^+^ monocytes using IL-4, granulocyte-macrophage colony-stimulating factor, and IL-10 or monophosphoryl lipid A, respectively. For KO experiments, CD4^+^ T cells were rested for 72 hours after electroporation in T cell media supplemented with recombinant human IL-2 (rhIL-2) (50 U/ml; PeproTech, Thermo Fisher Scientific, Waltham, MA, USA), then collected, and plated 10:1 with allogeneic DC-10s in 24-well plates at a concentration of 7 × 10^5^ to 1 × 10^6^ T cells/ml in T cell media with rhIL-10 (10 U/ml; Gibco, Thermo Fisher Scientific). For CRISPRa experiments, naïve CD4^+^ T cells were rested for 4 hours after electroporation in T cell media, washed, and plated 10:1 with allogeneic DC-10 in multiwell flat-bottom plates at a concentration of 7 × 10^5^ to 1 × 10^6^ T cells/ml in T cell media with rhIL-10 (10 U/ml; Gibco). For both workflows, half of the medium volume was replaced at day 5 of coculture with T cell media supplemented with rhIL-10 for a final concentration of 10 U/ml. Medium was replaced with base T cell media as necessary up until day 10 of coculture, at which point cells were collected.

### T cell anergy

T cell anergy assay was performed as described (*7*). Briefly, T-allo10 cells or control T-allo T_eff _ cells were labeled with CellTrace carboxyfluorescein succinimidyl ester (CFSE; Thermo Fisher Scientific) for 5 to 8 min at RT and cultured alone or stimulated with allogenic or third party matDCs at 10:1 ratio and 20:1 ratio of Human T-Activator CD3/CD28 Dynabeads (Thermo Fisher Scientific) for 3 days. After culture, the cells were collected and stained for viability and surface markers; the percentage of CFSE^−^, dividing cells within the live singlet CD3^+^CD4^+^ population, was measured by flow cytometry; and the anergy was calculated as [(% CFSE^−^ T-allo − % CFSE^−^ T-allo10)/% CFSE^−^ T-allo] × 100.

### Degranulation assay

To assess degranulation, 100,000 to 200,000 T-allo10 cells were cultured in a 96-well plate for 6 hours in total of 200 μl of the base T cell medium (see Primary Cells section) with brefeldin A (3 μg/ml; Sigma-Aldrich), 2 μM monensin (BD), and 5 μl of BV421 mouse anti-human CD107a antibody (clone H4A3, BD Biosciences), with or without anti-CD3/anti-CD28 Dynabeads or U937 myeloid leukemia cell line in 10:1 cell/bead ratio for 6 hours at 37°C and 5% CO_2_. After the culture, supernatant was removed by centrifugation, and cells were stained for surface and intracytoplasmic antigens using the protocol above and acquired. Antibodies for the degranulation panel are listed in table S17.

### T cell suppression

Autologous, responder CD4^+^ T cells (2.5 × 10^4^ per well), labeled with 5 μM CellTrace Violet (CTV; Thermo Fisher Scientific) for 5 to 8 min at RT, were cocultured with or without equal numbers of CFSE-labeled T-allo10 cells and stimulated with 2.5 × 10^3^ allogeneic matDCs for 5 days as described (*7*). Both responder and T-allo10 cells were also plated alone and with anti-CD3/anti-CD28 Dynabeads (Gibco) as proliferation controls. At day 5, the percentage of divided CTV^−^ responder cells was assessed by flow cytometry. Suppression was calculated as follows: % suppression = [(% divided responder cells with only matDCs added) − (% divided responder cells with T-allo10 and matDCs added)]/(% divided responder cells with only matDCs added).

### Intracellular cytokine staining

To assess intracellular IL-10 production, 100,000 to 200,000 T-allo10 cells were cultured in a 96-well plate for 6 hours in total of 200 μl of the base T cell medium (see the “Primary cells” section) Falcon tubes with brefeldin A (10 μg/ml) and 2 μM monensin ± phorbol 12-myristate 13-acetate and ionomycin (0.75 μg/ml; Sigma-Aldrich) for 6 hours at 37°C and 5% CO_2_. After washing, cells were stained for surface antigens and then intracellular IL-10 as described in the “Flow cytometry” section.

### Fluorescence-activated cell sorting

Purification of T-allo10 and T-allo cell populations, ex vivo CD4^+^ T cells, and ex vivo Tr1 cells was performed as described (*7*). Briefly, to isolate Tr1 cells and other subsets from T-allo10 cultures, cells were collected at day 10 of culture and stained as described (see the “Flow cytometry” section) using azide-free staining buffer. Samples were then filtered through a 40-μm cell filter (BD). Cells were sorted using a BD FACSAria II with a 100-μm nozzle by gating through singlets, live, lymphocyte, CD3^+^CD4^+^, CD45RA^−^ (memory) and lastly LAG3^+^CD49b^+^ for T-allo10 Tr1 cells; LAG3^−^CD49b^−^ for T-allo10 DN cells; and LAG3^−^CD49b^−^ for T-allo T_eff _ cells. To isolate total CD4^+^ T cells, cells were gated through singlets, live, lymphocyte, and CD3^+^CD4^+^ gate. To isolate naïve CD4^+^ T cells, cells were gated through singlets, live, lymphocyte, CD3^+^CD4^+^, and CD45RA^+^ gate.

### Sample processing and library generation for sc-immune profiling (RNA- and TCR-seq)

Live singlet CD3^+^CD4^+^ T cells from T-allo10 cell products were purified by FACS and counted, and their cell concentration was adjusted in PBS with 0.04% bovine serum albumin (BSA) to allow capture up to 10,000 cells/well of the Chromium Next GEM chip, which was then loaded onto a Chromium Single Cell Instrument (10x Genomics, Pleasanton, CA). RNA-seq and V(D)J libraries were prepared using the Chromium Single Cell 5′ Library, Gel Beads, and Multiplex Kit according to the manufacturer’s instructions. Paired-end sequencing was conducted on an Illumina NovaSeq 6000. Cell Ranger Single-Cell Software Suite (10X Genomics) was used for sample demultiplexing, alignment, filtering, and UMI counting for RNA-seq libraries using the human GRCh38 genome assembly and RefSeq gene model. For V(D)J libraries, the Cell Ranger Single-Cell Software (10X Genomics) was used for sample demultiplexing, de novo assembly, alignment, annotation against germline segment V(D)J reference sequences, and clonotype grouping.

The scRNA-seq data were processed using Seurat (v.3.0) with specific criteria: nFeature_RNA between 500 and 5000, percent.mito < 0.25, and nCount_RNA < 50,000. Cells with multiple TCR alpha or beta chains were filtered out. The filtered data were integrated, batch effect–corrected, clustered, and analyzed using the standard dataset integration and analysis workflow in Seurat. After merging samples into a single Seurat object, gene expression counts underwent normalization and scaling. Graph-based clustering was performed with the top 20 principal components at a resolution of 0.5. Marker genes for each cluster were identified, and clusters expressing canonical marker genes from different cell types were manually annotated. Differential gene expression analysis between T cell populations was conducted using Seurat FindMarkers() function.

TCR-seq data were analyzed using scRepertoire (v.1.0.2). A TCR clonotype was defined as the combination of genes in the TCR and the nucleotide sequence of the CDR3 region (gene + nucleotide) for paired TCR alpha and beta chains. Overlay of the position of clonally expanded cells was made with clonalOverlay(). To look at the network interaction of clonotypes shared between clusters along the single-cell dimensional reduction, we used the chord diagrams from the circlize R package using functions getCirclize() and chordDiagram(). In addition, the count of cells by cluster assigned into specific frequency ranges was obtained with the occupiedscRepertoire() function, and the clonal diversity was calculated using clonalDiversity(). To identify clonally related TCR sequences, clonal clustering was performed using the clonalCluster() function. This function groups TCR sequences based on sequence similarity using the normalized Levenshtein distance, which calculates the distance between two sequences and normalizes it by the mean of their lengths. Clustering was applied separately for the TCR alpha (TRA) and beta (TRB) chains using their amino acid sequences (sequence = “aa”). A similarity threshold of 0.85 was used to define connected sequences in the network, where sequences with a normalized edit distance below this threshold were assigned to the same clonal cluster. Unconnected sequences were assigned NA values. The resulting clonal clusters were appended to the metadata as TRA_cluster and TRB_cluster, which were mapped back to the original cell cluster labels and donor metadata. This allowed for the evaluation of clonal sharing across cell states and donors based on sequence similarity.

### Analysis of expression dynamics

We used SCANPY, a python package for large-scale differential gene expression analysis (https://github.com/scverse/scanpy) (*97*) to load our 10x output as anndata object. Structuring the data within an anndata matrix allowed for downstream compatibility with other python-based implementation methods. Preprocessing, filtering, clustering, and initial visualizations were performed using SCANPY, followed by RNA velocity analysis using scVelo (https://github.com/theislab/scvelo) (*98*). Velocyto, a command line interface (https://github.com/velocyto-team/velocyto.py) (*50*), was used to prepare the data to be analyzed in scVelo by generating a spliced/unspliced expression matrix that is necessary for estimating RNA velocities. Vector fields were plotted using scVelo’s velocity_embedding_grid function to visualize expression dynamics across all genes. To compare velocity and expression values of key Tr1 genes, the velocity function was used to plot the ratio of spliced and unspliced transcript as well as UMAP plots with colored gradient values of velocity and expression.

### Bulk RNA- and ATAC-seq sample preparation and sequencing

T-allo10 and control T-allo cells were generated from total CD4^+^ T cell/dendritic cell donor pairs generated from five healthy donors as previously described [(*7*) and Fig. 2A]. Tr1 and non-Tr1 cells were isolated from T-allo10 cell products, and T_eff_ cell isolated from T-allo cell products at day 10 of culture by FACS as described above. Along with an aliquot of previously cryopreserved CD4^+^ T cells, these populations were either lysed with RNAqueous lysis buffer, after which the RNA was isolated and libraries prepared as described (*7*), or their native DNA was tagmented and libraries prepared following the OMNI-ATAC protocol (*32*), with two modifications; transposition reaction was done with 350-rpm mixing and using the transposase from the Illumina Tagment DNA TDE1 Enzyme and Buffer Kit (Illumina, San Diego, CA, USA). Libraries were quantified using a KAPA Library Quantification kit (Roche, Indianapolis, IN, USA) and then sequenced using HiSeq 4000 sequencer (Illumina).

For bulk ATAC-seq of CD4^+^ T cells, CD4^+^ T cells were isolated using FACS from cryopreserved PBMC from the first cohort of patients with hematological malignancies infused with 1 × 10^6^ T-allo10 cells/kg a day before their unmanipulated allo-HSCT (ClinicalTrials.gov ID: NCT03198234) at days 35, 90, and 300 posttreatment (*n* = 3) and four healthy donors. Patient characteristics have been described (*67*). The frequency of Tr1 cells in ex vivo CD4^+^ T cells was measured using flow cytometry (*7*) in patients and three of four of the healthy donors. ATAC libraries were made from live purified CD4^+^ T cells using OMNI-ATAC protocol and sequenced with NextSeq 500 sequencer (Illumina).

### Bulk ATAC-seq data analysis

Adapters were trimmed using cutadapt, and reads were mapped to the hg38 genome using bowtie2 with a maximum fragment length of 2000 base pair (bp). Filtering criteria included nonmitochondrial reads, mapq > 20, and properly paired reads. Duplicates were removed using Picard tools. Peak calling was performed using macs2 with specific parameters (--shift 75 --extsize 150 --nomodel --call-summits --nolambda -p 0.01 -B –SPMR) on Tn5 insertion sites.

ChrAccR (https://github.com/GreenleafLab/ChrAccR) was used for differential accessibility analysis between sorted T cell populations. Regions and fragments on the non-autosomal chromosomes (mitochondrial, X, and Y) and regions with a coverage of less than 1 in more than 50% of samples were removed. A count matrix was generated as insertion counts across samples at consensus peakset regions using the ChrAccR regionAggregation() function. DESeq2 was used for calculating differentially accessible peaks and visualized using MA plots from the ggmaplot package. TF motif enrichment was calculated using ChromVAR (*34*), and deviation scores were obtained with the ChrAccR getChromVarDev() function. Footprint plots were generated using aggregations of insertion counts in windows surrounding all occurrences of a motif genome wide. ChrAccR normalizes these counts using *k*-mer frequencies and uses all insertion sites to calculate the background distribution. Differential footprint analysis was performed with TOBIAS (*35*) using the function BINDetect.

We used merged peak files to visualize peak accessibility in the WashU Epigenome Browser (http://epigenomegateway.wustl.edu). The differential accessibility between Tr1 and the other CD4 T cells subsets was calculated as described previously, focusing on peaks overlapping with TF promoter and enhancers regions predicted by TRANSFAC 2.0 database.

### Bulk RNA-seq data processing and analysis

Bulk RNA-seq was processed as described (*7*). Briefly, sequencing reads were mapped and aligned to the reference human genome (GENCODE v32) and quantified using Salmon (v1.10.2), then aggregated to gene level, and imported into R using tximport. Samples were filtered to remove reads with low or no expression across multiple samples. Normalization, visualization, and differential gene expression analysis were performed using DESeq2 package (*99*). The integration of RNA and ATAC data was accomplished through a paired analysis of corresponding donors. This involved identifying and examining the correlation between highly expressed genes in the RNA dataset and accessible genes in the ATAC dataset using an adaptation of functions of the FigR (Functional Inference of Gene Regulation) package (v 0.1.0)

### Upstream regulator identification using ingenuity pathway analysis

We annotated the observed peaks in the bulk ATAC-seq dataset of Tr1 and control cells according to the nearest gene, obtaining 16,391 hits. Because multiple peaks often are associated to the same gene, we used gene pattern module of GSEA (*36*) to collapse the gene list by ranking them using each gene’s differential expression between Tr1 cells and control populations. First, gene accessibility profiles were obtained from ATAC-seq data. The samples were divided into two groups: the Tr1 cells and the rest of the T cell populations combined. GSEA was used to identify genes that were significantly enriched or depleted in the Tr1 cells versus the rest and ranked accordingly. We selected genes ranked ≥0.5 in Tr1 cells (range: −0.8 to 1.95) to upload into IPA and perform Upstream Regulator analysis. Results of the Upstream Regulator analysis were further filtered to select molecules that are: (i) significantly enriched (*P* < 0.05), (ii) expressed in the analyzed dataset, and (iii) annotated by IPA as transcriptional regulators (i.e., TFs).

### Functional analysis of cis-regulatory regions

The GREAT (*39*) gene ontology tool (v.4.0.4, http://great.stanford.edu/public/html/index.php) was used to assign Gene Ontology terms to cis-regulatory regions with high accessibility in the Tr1 cluster (Tr1 peaks were defined as those with FDR < 0.01 and log2 fold change > 1.5 compared with other CD4^+^ T cell populations). Parameters used were as follows: species assembly: hg38, association rule: basal + extension; 5000-bp upstream, 1000-bp downstream, 1,000,000-bp max extension, and curated regulatory domains included. We also used FigR package (v.0.1.0; https://github.com/buenrostrolab/FigR) to discern cis-regulatory interactions and delineate DORC. For visualization, we used the function dorcJPlot().

### CRISPR-Cas9 knockouts

sgRNAs were purchased from Synthego (Menlo Park, CA, USA). The sgRNAs were modified to incorporate 2′-*O*-methyl-3′-phosphorothioate bonds at the three terminal nucleotides of the 5′ and 3′ ends (*100*). For each gene, one to three sgRNAs were selected using CRISPOR (*101*) and used in combination. HiFi Cas9 was purchased from Integrated DNA Technologies (IDT; Coralville, IA, USA). When starting from cryopreserved CD4^+^ T cells, cells were thawed and rested in an 37°C and 5% CO_2_ incubator in the T cell medium with rhIL-2 (50 U/ml; PeproTech) for 4 to 24 hours before electroporation, while freshly isolated CD4^+^ T cells were electroporated immediately following isolation. A total of 37.2 pmol of Cas9 and 105 pmol of sgRNA were incubated for 30′ at room temperature to form the ribonucleoprotein (RNP) complex. T cells were resuspended in P3 buffer and mixed with RNPs and 10 pmol of Cas9 Electroporation Enhancer (IDT). Cells were electroporated using the Lonza 4D Nucleofector X with program EO-115 and then plated at 1 × 10^6^ to 4 × 10^6^ cells/ml in T cell media with rhIL-2 (50 U/ml) and rested for 72 hours before lysis for Sanger sequencing analysis or T-allo10 culture initiation. *IRF4* KO experiments had initially control parental CD4^+^ T cells that were mock-treated (nucleofected only), which was then reproduced with nontargeted sgRNAs; since there was no statistically significant difference between the two control conditions, all experiments were included. CRISPR-Cas9 KO sequences are listed in table S18.

### Indel frequency analysis

Sixty to 72 hours posttargeting, CD4^+^ T cells were collected and guide DNA (gDNA) was isolated with Quick Extract (Lucigen) following the manufacturer’s instructions. The following PCR primers were used to amplify cut sites with Q5 Hot Start High-Fidelity 2X Master Mix (New England Biolabs, Ipswich, MA, USA): IRF4 (5′-AGGTGCCTTCTTCCGGGG-3′; 5′-TTGCGTGGAAACGAGAACGC-3′), BATF ( 5′-GAAGTTTCCGCCCATGTGAC-3′; 5′-CGGCCCACTTGAAAACTCCT-3′), and MAF (5′-CGCCGCGCAAGCTAGAA-3′; 5′-GGGTAGCCGGTCATCCAGT-3′). PCR products were Sanger sequenced using one of the primers above. Resulting Sanger chromatograms were then used as input for indel analysis (Synthego Performance Analysis, ICE Analysis. 2019. v3.0. Synthego; February 2023).

### CRISPR-activation

Chemically modified sgRNAs for CRISPRa were selected using CRISPick (https://portals.broadinstitute.org/gppx/crispick/public) (*102*). Two guides were chosen on the basis of guanine-cytosine content and proximity to transcription start site (TSS) and then used in combination. sgRNAs, with chemical modifications identical to those used for CRISPR-Cas9 knockouts, were purchased from Synthego. dead (d) Cas9-VPR (VP64, p65, Rta) mRNA was obtained from Horizon Discovery (Waterbeach, UK). Cells were resuspended in P3 buffer (Lonza) and mixed with 105 pmol of sgRNA and 0.6 μg of dCas9-VPR mRNA, followed by electroporation on a Lonza 4D Nucleofector X unit with program EO-115 using a Lonza electroporation strip with no more than 1.5 × 10^6^ cells per well. Cells were plated at 1 × 10^6^ to 4 × 10^6^ cells/ml in T cell media and rested before lysis for qPCR and T-allo10 culture initiation. CRISPRa guide sequences are listed in table S18.

### ELISA

IL-10 enzyme-linked immunosorbent assay (ELISA) was performed as described (*7*).

### RNA isolation and qPCR

Twenty-four to 48 hours after the electroporation of CRISPRa components, cells were collected and lysed using RNeasy Micro Kit RLT buffer (QIAGEN, Hilden, Germany) and then passed through Chromatrap gDNA removal columns (Porvair Sciences, King’s Lynn, UK). From here, QIAGEN RNeasy Micro Kit instructions were followed for RNA extraction minus deoxyribonuclease treatment steps. RNA yield was quantified using a Qubit 4 fluorometer (Invitrogen, Thermo Fisher Scientific), and reverse transcription was carried out using SuperScript VILO IV mastermix (Invitrogen). The cDNA input for quantitative polymerase chain reaction (qPCR) was normalized between samples based on pre-cDNA synthesis RNA concentrations. Taqman Fast Universal PCR master mix and Taqman gene expression assays (Applied Biosystems, Thermo Fisher Scientific) were used for qPCR. The assay IDs of the Taqman assays used are as follows: HS00943570_m1 for RUBCN and Hs_00193519_m1 for MAF. qPCR was performed on a QuantStudio 7 Pro (Applied Biosystems) and analyzed using Design and Analysis v2.6 software (Thermo Fisher Scientific). qPCR was run with three technical replicates per sample. Target transcript abundance was normalized to endogenous control (RUBCN) abundance within samples before assessing CRISPRa efficiency by comparing nontargeted and targeted conditions.

### Estimation of Tr1 cell abundances with CIBERSORTx

The CIBERSORTx (*66*) algorithm was employed to generate a signature matrix of accessible motifs for isolated Tr1 cells (Create Signature Matrix module), which involved analyzing motif deviations from bulk ATAC-seq data of isolated CD4^+^ T cells, Tr1 cells, and non-Tr1 (DN) cells from T-allo10 products. Subsequently, the generated signature matrix was applied to predict the composition of Tr1 cells within total CD4^+^ T cells from both healthy donors and post–allo-HSCT patients, as well as following T-allo10 infusion, using bulk ATAC data (Input Cell Fractions module).

### Sample processing and library generation for sc-multiome (ATAC- and RNA-seq)

CD4^+^ T cells were purified by FACS as for the bulk ATAC-seq. Nuclei were isolated from 40,000 cells according to the demonstrated protocol: nuclei isolation for single cell multiome atac + gene expression sequencing (10x Genomics, CG000365). In summary, cells were washed twice by centrifugation with PBS with 0.04% BSA (Miltenyi Biotec) and then lysed with 45 μl of chilled lysis buffer. After 3.5-min incubation on ice, the lysate was washed with 50 μl of chilled wash buffer, supernatants were removed, and pellets were washed again without disruption by adding 45 μl of chilled Diluted Nuclei Buffer to each tube. Nuclei were then resuspended in 7 μl of diluted nuclei buffer, and a 2 μl of aliquot was used for manual counting with hemocytometer and 0.4% Trypan Blue solution. After counting, nuclei concentration was adjusted to achieve maximum targeted nuclei recovery and promptly processed for single-cell library generation using the manufacturer’s protocol. Libraries were prepared according to the Chromium Next GEM Single Cell Multiome ATAC + Gene Expression User Guide (CG000338 Rev F). Paired-end 150-bp sequencing was done either on NexSeq 500 using the v2.5high-output kit (sample from healthy donor 10242) or using Illumina NovaSeq 6000, targeting a depth of 250 million read pairs per sample. More than 5000 nuclei were captured per donor, and samples were passed the quality control postsequencing (table S19).

Demultiplexed scRNA- and scATAC-seq fastq files were processed using the Cell Ranger ARC pipeline (version 2.0.0) from 10x Genomics, generating barcoded count matrices for gene expression and ATAC data. The R package ArchR (*60*) (v 1.0.1) was used for downstream analysis. In ArchR, count matrices for each sample were imported and filtered for barcodes present in both scRNA-seq and scATAC-seq datasets. ArchR quality control included filtering for nuclei with 200 to 50,000 RNA transcripts, <1% mitochondrial reads, <5% ribosomal reads, TSS enrichment >4, and >1000 ATAC fragments. Subsequently, the filterDoublets() function in ArchR automatically removed doublets by identifying and eliminating the nearest neighbors of simulated doublets. For visualization and cluster identification, we first identify variable peaks and perform dimensionality reduction using the ArchR addIterativeLSI() function. When the latent semantic indexing (LSI) approach is not enough to correct strong batch effect differences, ArchR implements a commonly used batch effect correction tool called Harmony with the function addHarmony(). For visualization, we used UMAP embeddings using the addUMAP() function with 30 neighbors. Clustering was performed using the addClusters() function with resolution parameters ranging from 0.4 to 1.2 to identify cell populations at varying granularity. For scRNA-seq data processed with Seurat, we performed neighborhood graph construction using FindNeighbors() on the first 20 principal components, followed by clustering with FindClusters() at a resolution of 0.5. To do cell-type annotation, we use prior knowledge of cell type–specific marker genes and data-driven annotation tool CIPR (*44*). Clusters were further refined by integrating gene expression, motif activity (via ChromVAR), and chromatin accessibility; cells displaying markers for non-CD4^+^ T cell lineages, such as CD8^+^ T cells or NK cells, were identified and excluded from downstream analysis. Gene expression was estimated from chromatin accessibility data using ArchR gene scores. Pseudo-bulk ATAC replicates were created for each cell type, and chromatin accessibility peaks were called using MACS2. Marker peaks were identified using Wilcoxon pairwise comparisons using ArchR getMarkerFeatures. For motif analysis, ArchR also calculates motif deviations using ChromVAR. TF motif enrichment analysis on scATAC peaks was executed using the peakAnnoEnrichment() function in ArchR. Default parameters were used using position frequency matrices from cis-BP. The differential analysis between clusters was conducted using the getMarkerFeatures() function within the GeneScoreMatrix (for accessibility), GeneExpressionMatrix, and MotifMatrix.

### Trajectory analysis of Tr1 cell differentiation using ArchR

To investigate the dynamics of gene expression and identify upstream regulators during Tr1 cell differentiation, we used the ArchR platform for trajectory analysis. ArchR allows for the ordering of cells in pseudo-time by creating cellular trajectories within a lower N-dimensional subspace derived from an ArchRProject. This approach enables the precise alignment and ordering of cells along a biologically relevant trajectory, facilitating the study of dynamic processes such as differentiation. We first defined a trajectory backbone based on known day of cell culture corresponding to different stages of Tr1 differentiation, and a pseudo-time vector was assigned to each cell along the trajectory. By aligning all cells to the continuous trajectory and scaling the pseudo-time to a 100-point scale, ArchR facilitated downstream analyses, including the identification of key TFs and signaling pathways involved in Tr1 differentiation.

### ccRCC scATAC data processing and re-analysis

The fragment files corresponding to each patient from the scATAC-seq dataset published in (*73*) were obtained from the National Center for Biotechnology Information Gene Expression Omnibus (GEO) repository under accession number GSE181062 and analyzed in the same manner as above. ArchR was used for the analysis of total CD45^+^ cells from all patients. Potential doublets were identified and subsequently removed, followed by batch correction using the Harmony. Following dimensionality reduction and clustering, the cell clusters corresponding to *CD20* (MS4A1)^+^ B cells, CD14^+^ monocytes, and NCAM1^+^/FCGR3A^+^ NK cells were excluded from downstream analysis, focusing exclusively on T cells. Specifically, T cell clusters were identified on the basis of the expression of *CD3D* and *CD3E* markers. Subsequently, the CD8^+^ T cell subset was selectively removed, retaining only the CD4^+^ T cell population for further analysis.

### Tr1 cell identity prediction using ScType

ScType computational platform, specifically designed for cell-type identification in scRNA-seq datasets, was used to predict the identity of Tr1 cells in CD4^+^ T cells and solid tumors from single-cell data. The scRNA-seq data used for the analysis were preprocessed to include only high-quality cells, followed by normalization and scaling using standard procedures. Genes with low expression across cells were filtered out to reduce noise, and the data were log-normalized to stabilize variance. The preprocessed scRNA-seq data were input into the ScType, which operates by comparing the expression profiles of individual cells against a comprehensive reference marker genes of non-Tr1 cells and Tr1 markers of cell identity (table S20). For each cell, ScType calculates a “cell score,” which quantifies the similarity of the cell’s gene expression profile to known cell types in the refence genes. The cell score, generated by ScType, was used to predict the identity of Tr1 cells clusters.

### Reanalysis of published single-cell datasets

Publicly available single-cell datasets from various studies were reanalyzed:

1) Advanced TNBC: scRNA-seq data were obtained from a study examining the combination therapy of paclitaxel and atezolizumab in 22 patients with advanced TNBC. The dataset includes transcriptomes profiles of CD45^+^ immune cells from primary/metastatic tumor tissues (*76*).

2) ccRCC: This dataset comprises scRNA-seq (5′ RNA expression sequencing) data from immune cells collected from the blood, normal adjacent kidney, and malignant tissues of patients with early-stage ccRCC at baseline (*73*).

3) CRC: scRNA-seq data from T cells of patients with CRC were obtained, where cells were sorted and profiled using the Smart-seq2 protocol (*74*).

4) Mouse sarcoma: This dataset comprises scRNA-seq data from a murine sarcoma model. It characterizes the phenotypic and functional shifts in tumor-specific CD4^+^ T cells following vaccination with varying doses of major histocompatibility complex-II–restricted neoantigens. The study compares the generation of helper CD4^+^ T cells in low-dose vaccine (LD or LDVax) cohorts against the suppressive Tr1 cells in high-dose vaccine (HD or HDVax) cohorts.

The raw and processed sequencing data for each study were downloaded from public repositories (GEO accession numbers GSE169246, GSE181061, GSE108989, and GSE268301 respectively). For scRNA-seq datasets, the data were processed using standard Seurat pipelines to define cluster populations, differential gene expression, and prediction of Tr1 identity. When scTCR-seq was publicly available, we also calculated the frequency of clonal cells using clonalOccupy() function from scRepertertoire (v2.3.2).

### Statistical analysis

For in vitro experiments, sample sizes were not predetermined using statistical methods but were comparable to those in previous publications (*7*). Except in cases of technical failure or as indicated in fig. S10, no data were excluded from the analyses, and the experiments were not randomized. Investigators were not blinded during experiments or outcome assessment, and data collection and analysis were not performed blind to experimental conditions. Statistical analysis was carried out in GraphPad Prism 9 and 10 (GraphPad Software Inc., Boston, MA, USA) or R v.4.0.3. Data analyzed using GraphPad Prism used nonparametric tests that do not assume equal variances between groups: Mann-Whitney or Wilcoxon test for groups of two (unpaired or paired samples, respectively); Kruskal-Wallis or Friedman analysis of variance (ANOVA) with Dunn’s post hoc test for >2 groups (independent or dependent samples, respectively); and Spearman rank *r* for correlation, with testing level (α) of 0.05. Tests were two-sided unless otherwise indicated. As applicable, center bars and whiskers represent the median with interquartile range. The number of biological replicates (i.e., donors), *n*, is indicated in figure captions for each experiment. The R packages used in the analysis of sequencing data have embedded statistical algorithms that include multiple testing correction for *P* values. FDR of 0.05 was applied. All measurements displayed were taken from distinct samples, i.e., biological replicates.
