# Supplementary material for: Epigenetic landscape, key transcriptional regulators, and in vivo identification of human Tr1 cells
Source: Sci Adv. 2026 Jul 10;12(28):eaec6358. doi: 10.1126/sciadv.aec6358 (PMC13353415; doi:10.1126/sciadv.aec6358)
Supplement: Supplementary file 1 — Figs. S1 to S15 Legends for data S1 and S2 [file sciadv.aec6358_sm.pdf]

Science Advances
AAAS

# Supplementary Materials for

## Epigenetic landscape, key transcriptional regulators, and in vivo identification of human Tr1 cells

Alma-Martina Cepika *et al.*

Corresponding author: Alma-Martina Cepika, acepika@stanford.edu; Maria Grazia Roncarolo, mg1@stanford.edu

*Sci. Adv.* **12**, eaec6358 (2026)
DOI: 10.1126/sciadv.aec6358

**The PDF file includes:**

Figs. S1 to S15
Legends for data S1 and S2

**Other Supplementary Material for this manuscript includes the following:**

Data S1 and S2

# Supplementary Figure 1

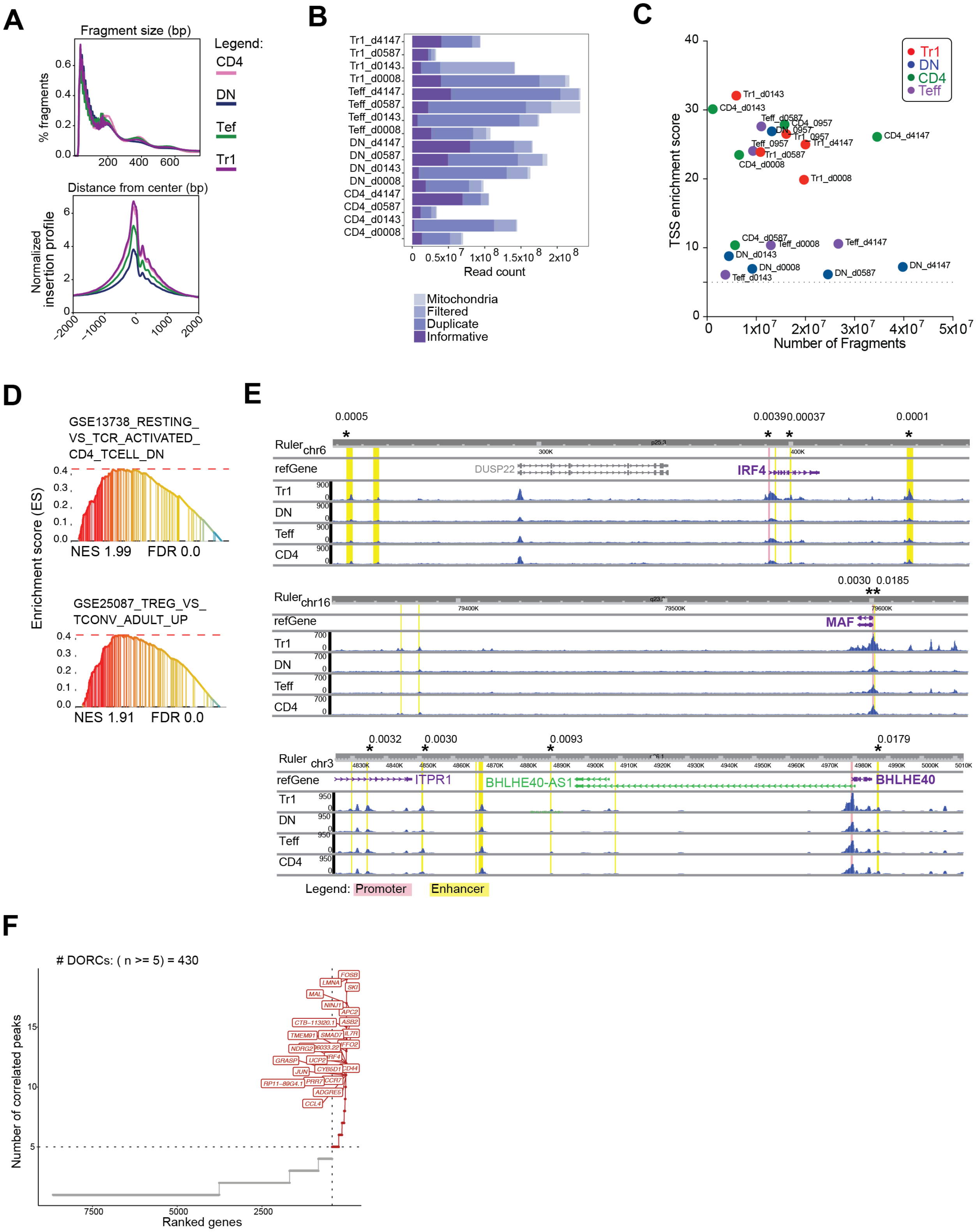

**Fig. S1. A.** Quality control analysis (QC) of bulk ATAC libraries showing nucleosome periodicity (left) and transcription start site enrichment (right). Data are averaged from n = 5 per condition. **B.** Sequence quality and unique alignment sequence distribution of the ATAC-seq dataset. **C.** ATAC-seq data quality control filters of four $CD4^+$ T cell populations from five donors. Shown are the numbers of unique ATAC-seq nuclear fragments and transcription start site (TSS) enrichment in each sample. Dashed lines represent the filters for high-quality data ($1 \times 10^6$ unique nuclear fragments and TSS score $\geq 5$). **D.** Gene Set Enrichment Analysis (GSEA) of ATAC-seq peaks of Tr1 cells versus all non-Tr1 cells within MsigDB C7: Immune Signatures database; representative enrichment plots are shown. NES = normalized enrichment score; FDR = FDR *q* value. **E.** Gene accessibility track visualization of transcription factors IRF4, MAF and BHLHE40. Promoter regions are highlighted in red, while enhancer regions are highlighted in yellow. Significantly accessible regions in Tr1 cells compared to the non-Tr1 cells are marked with asterisks (*), with corresponding *p*-value indicated numerically. **F.** Visualization of ranked genes based on the number of significant gene-peak correlations (per gene), used to define domains of regulatory chromatin (DORC).

# Supplementary Figure 2

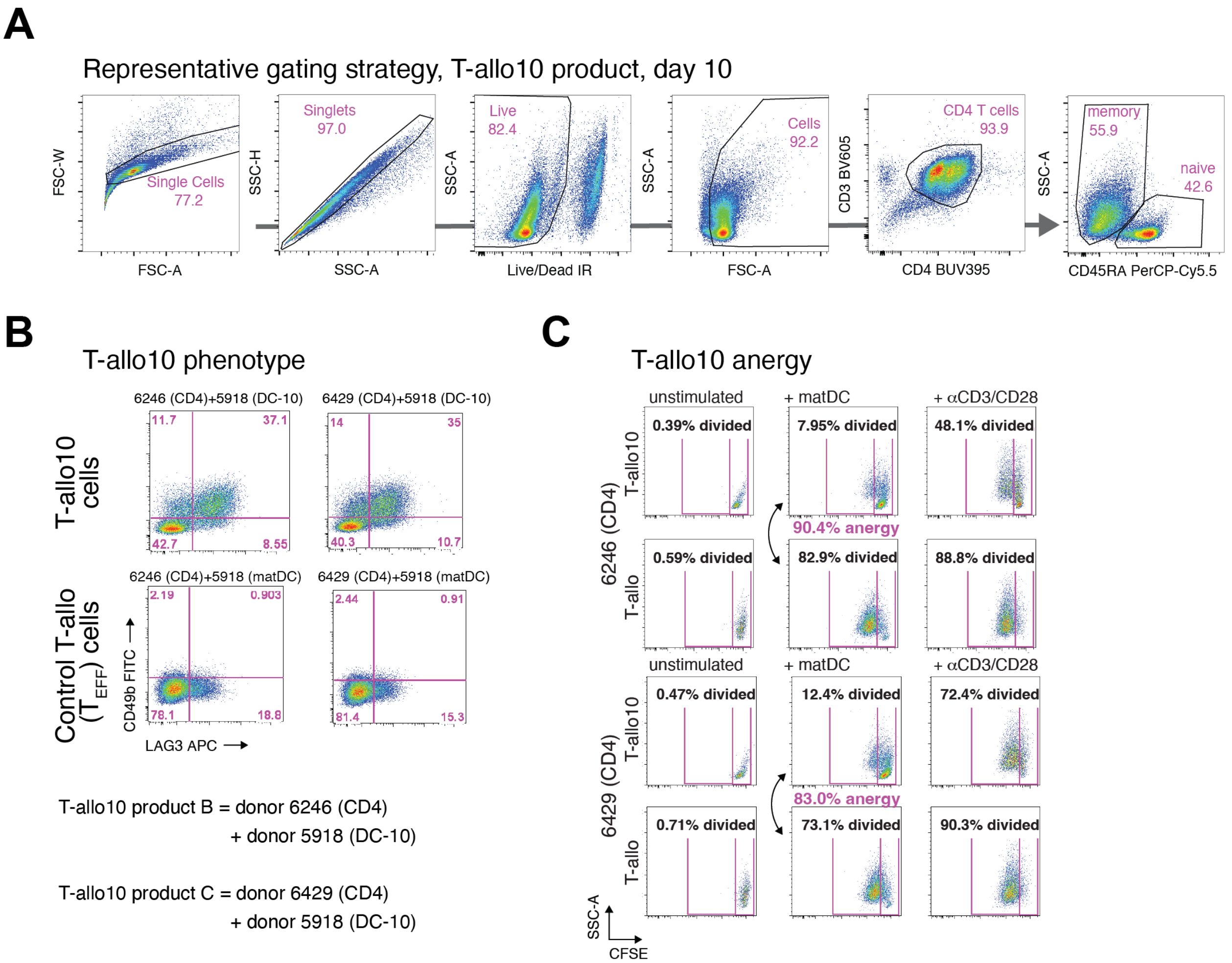


**Fig. S2. A.** Representative gating flow cytometry strategy of T-allo10 products. **B.** Percentage of Tr1 cells in two T-allo10 and control T-allo products, defined as $CD49b^{+}LAG3^{+}$ memory ($CD45RA^{-}$) $CD4^{+}$ $CD3^{+}$ live singlet T cells; flow cytometry. **C.** Alloantigen–specific anergy of two T-allo10 products analyzed by flow cytometry, measured as difference in proliferation of carboxyfluorescein diacetate, succinimidyl ester (CFSE)-labeled T-allo10 cells and control effector cells (T-allo) in response to mature dendritic cells (matDC). Polyclonal stimulation with plate-bound anti-CD3 and soluble anti-CD28 monoclonal antibody was used as a positive control[7].

# Supplementary Figure 3

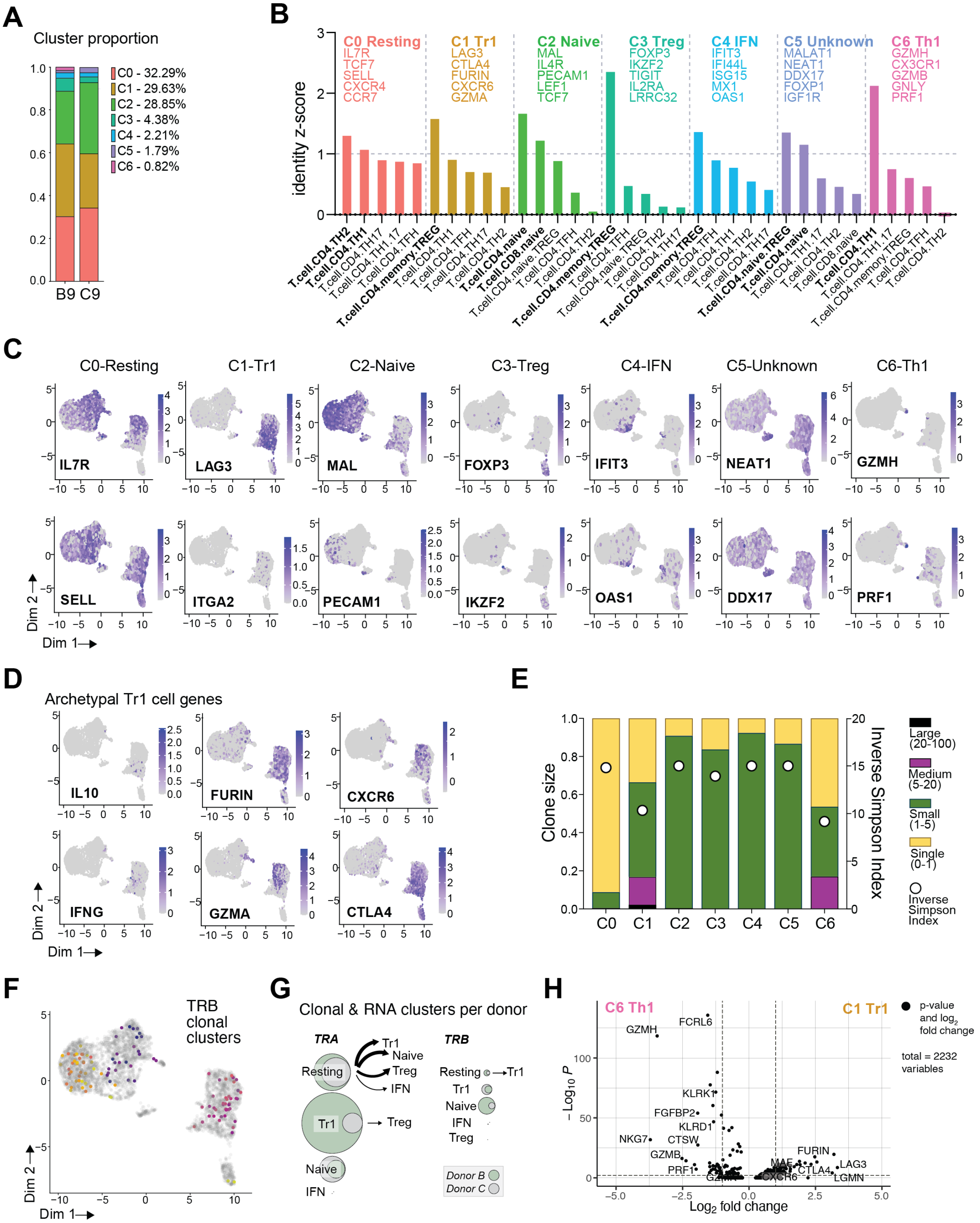

**Fig. S3. A.** Distribution of cluster fractions for each donor, showing the average of 2 donors. **B.** Clustering label annotation as predicted by: a) CIPR annotation tool (graph) and b) expression level of canonical markers of T cell subsets. **C.** Representative UMAP visualization of gene expression from each cluster's defining markers. **D.** UMAP representation of gene expression from canonical Tr1 marker genes. **E.** Clone size distribution of clusters and clonal diversity calculated by inverse Simpson index. **F**. UMAP visualization of TRB clonal clusters; cells with the similar CDR3 sequences by edit distance are represented with the same color. Cells in grey = no identified clusters. **G**. Distribution of cells with same clonal clusters across scRNA-seq clusters. Circle diameter is proportional to the number of cells with similar CDR3 sequence within one scRNA-seq cluster (min = 1, max = 173), and line thickness proportional to the number of cells with similar CDR3 sequence shared across scRNA-seq clusters (min = 1, max = 4). **H.** Volcano plot depicting the results of the differential gene expression analysis between Tr1 and Th1 clusters.

# Supplementary Figure 4

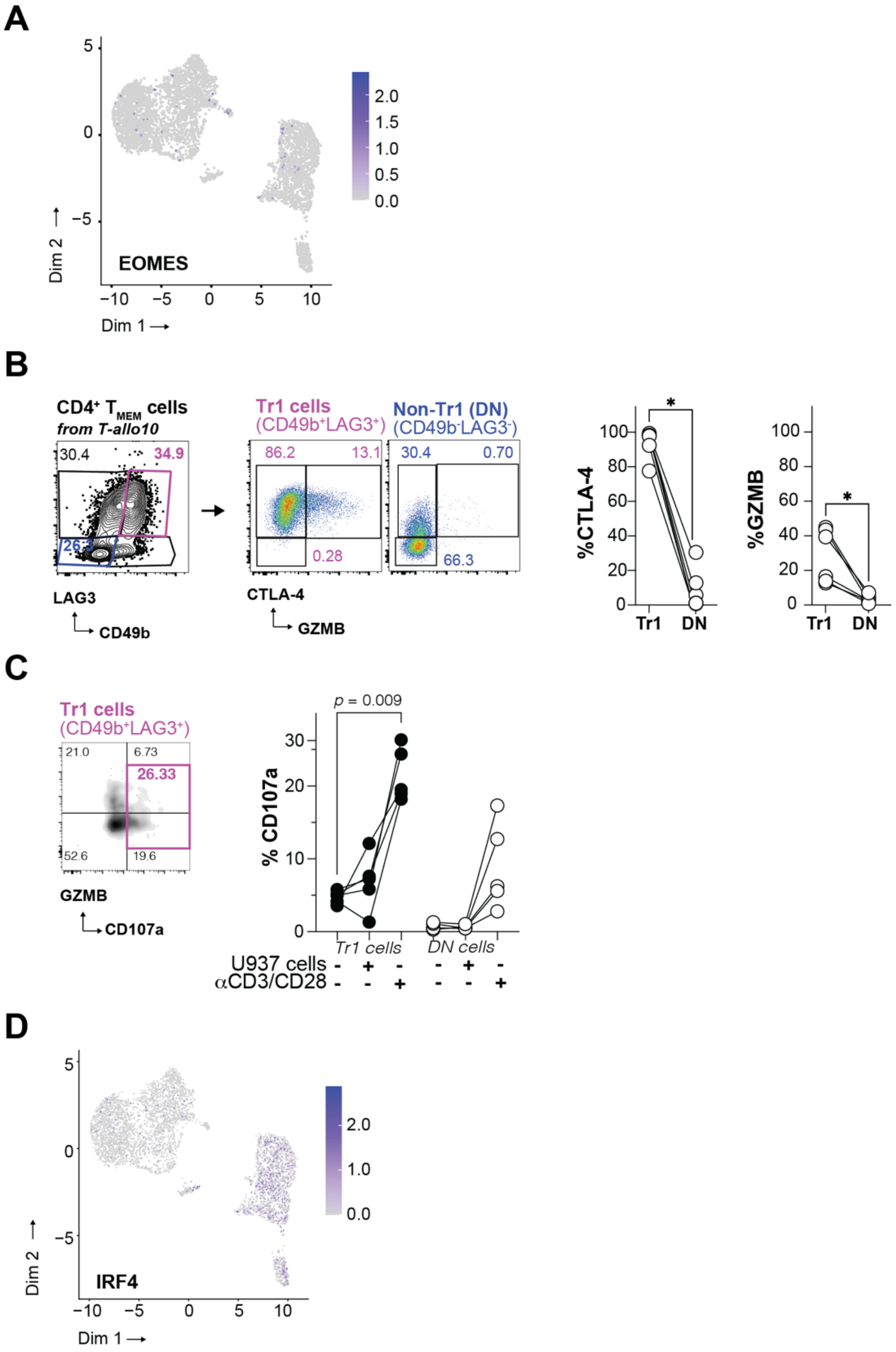

**Fig. S4. A.** UMAP visualization of EOMES gene expression in sc-immune profiling of two T-allo10 products. **B.** Left: representative plots; right: cumulative data, intracellular CTLA-4 and granzyme B (GZMB) expression in $CD49b^{+}LAG3^{+}$ Tr1 and $CD49b^{-}LAG3^{-}$ (DN) non-Tr1 memory $CD4^{+}$ T cells from T-allo10 products. Flow cytometry, n = 6. *$p < 0.05$, Wilcoxon test. **C.** Left: representative plots, and right: cumulative data showing the frequency of Tr1 cells or DN cells expressing intracellular granzyme B (GZMB) and surface CD107a, which indicates degranulating cells, after 6h-culture of T-allo10 products (n = 5) in cell culture media ± anti-CD3/CD28 Dynabeads or myeloid tumor cell line U937 cells in 10:1 ratio. Tr1 vs DN cells across conditions: Friedman ANOVA with Dunn's post hoc test. Tr1 vs DN cells in each condition: Wilcoxon test. Statistically significant adjusted *p*-values are indicated. **D.** UMAP visualization of IRF4 gene expression in sc-immune profiling of two T-allo10 products.

## Supplementary Figure 5

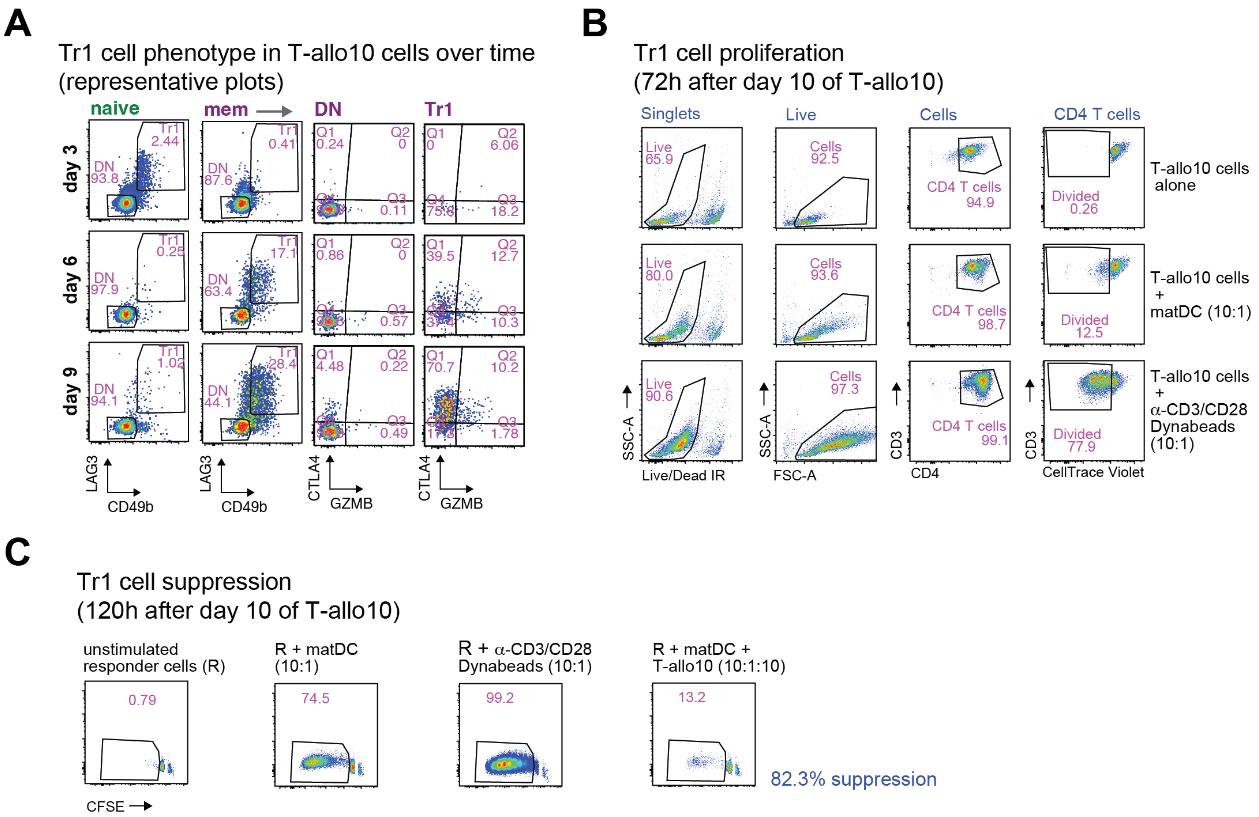


**Fig. S5. A.** Gating strategy for identifying Tr1 cell populations over time, highlighting key surface and intracellular markers used to define Tr1 cells at different stages of differentiation. **B.** Proliferation assay, showing the percentage of live, CellTrace Violet- proliferating T-allo10 cells after culture in medium only, re-stimulated with allogeneic mature dendritic cells (matDC) derived from the same DC donor used to generate T-allo10 cells (antigen-specific stimulus), and stimulated with anti-CD3/CD28 Dynabeads (non-specific stimulus) in indicated ratios. **C.** Suppression assay, demonstrating the functional ability of Tr1 cells to suppress the proliferation of syngeneic responder $CD4^{+}$ T cells (R) under indicated conditions (described in B). Suppression is calculated as outlined in **Methods**.

# Supplementary Figure 6

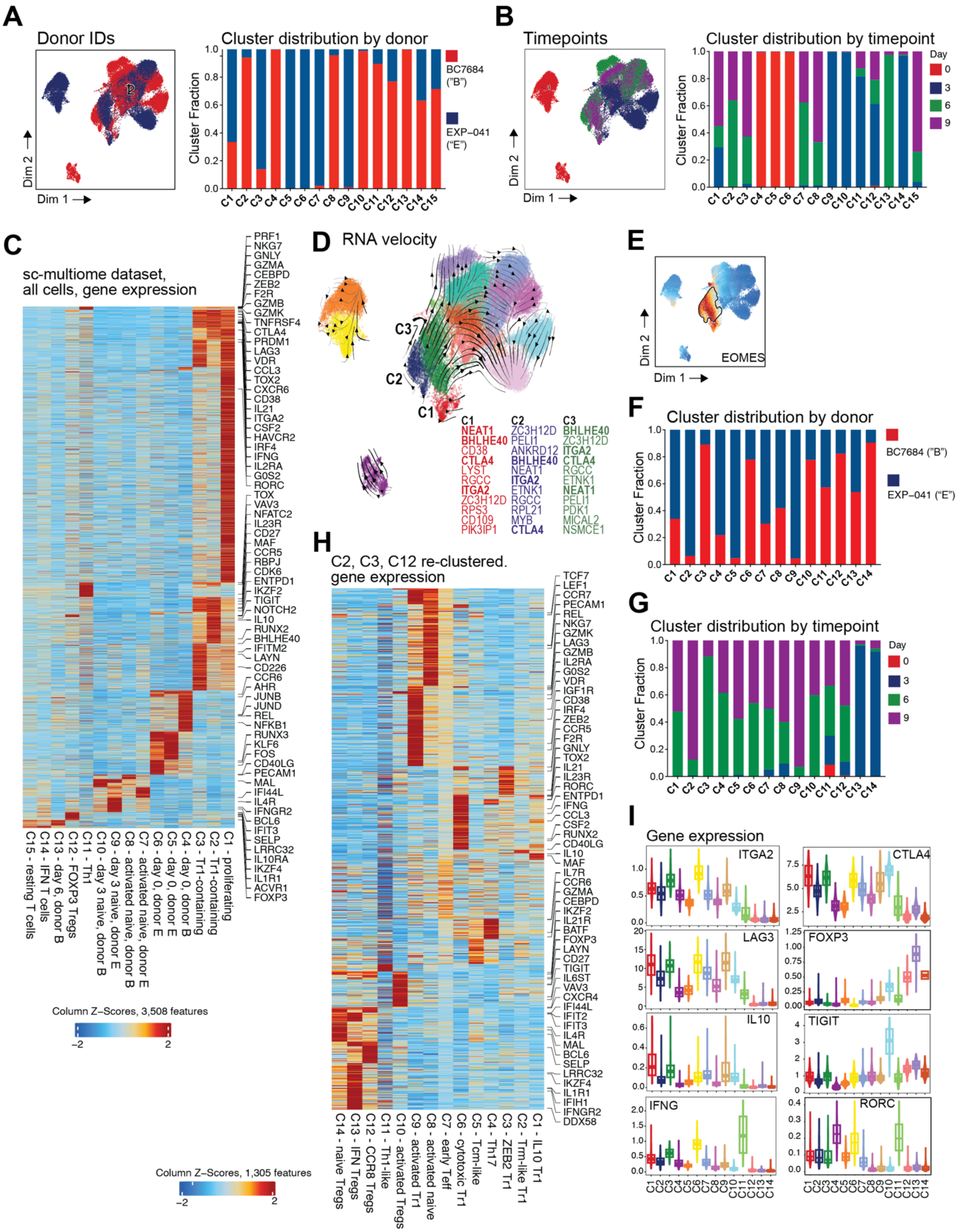

**Fig. S6. A.** UMAP plot (left) showing donor distribution across clusters, with the corresponding bar plot (right) illustrating the proportion of cells from each donor per cluster. Longitudinal sc-multiome analysis of Tallo10 cells. **B.** UMAP plot (left) depicting the distribution of samples by time point during culture, overlaid on cell clusters, and a bar plot (right) summarizing the proportion of cells from each time point per cluster. **C.** Heatmap illustrating gene accessibility across clusters. **D.** RNA velocity analysis, with highlighted proliferating, activated cell cluster C1 and Tr1-enriched clusters C2 and C3; arrows indicate the inferred direction of cell fate transitions. **E.** UMAP representation of EOMES expression levels across clusters. **F.** Bar plot of donor distribution per sub-cluster; analysis of re-clustered FOXP3$^{+}$ Treg (C12) and Tr1-enriched cells (C2, C3). **G.** Bar plot of time-point distribution per sub-cluster; analysis of re-clustered FOXP3$^{+}$ Treg and Tr1 cells. **H.** Heatmap of gene expression for re-clustered Tr1 and FOXP3$^{+}$ Treg cells, identifying distinct transcriptional profiles. **I.** Gene expression levels of marker genes used for sub-cluster annotation of re-clustered Tr1 and FOXP3$^{+}$ Treg cells.

# Supplementary Figure 7

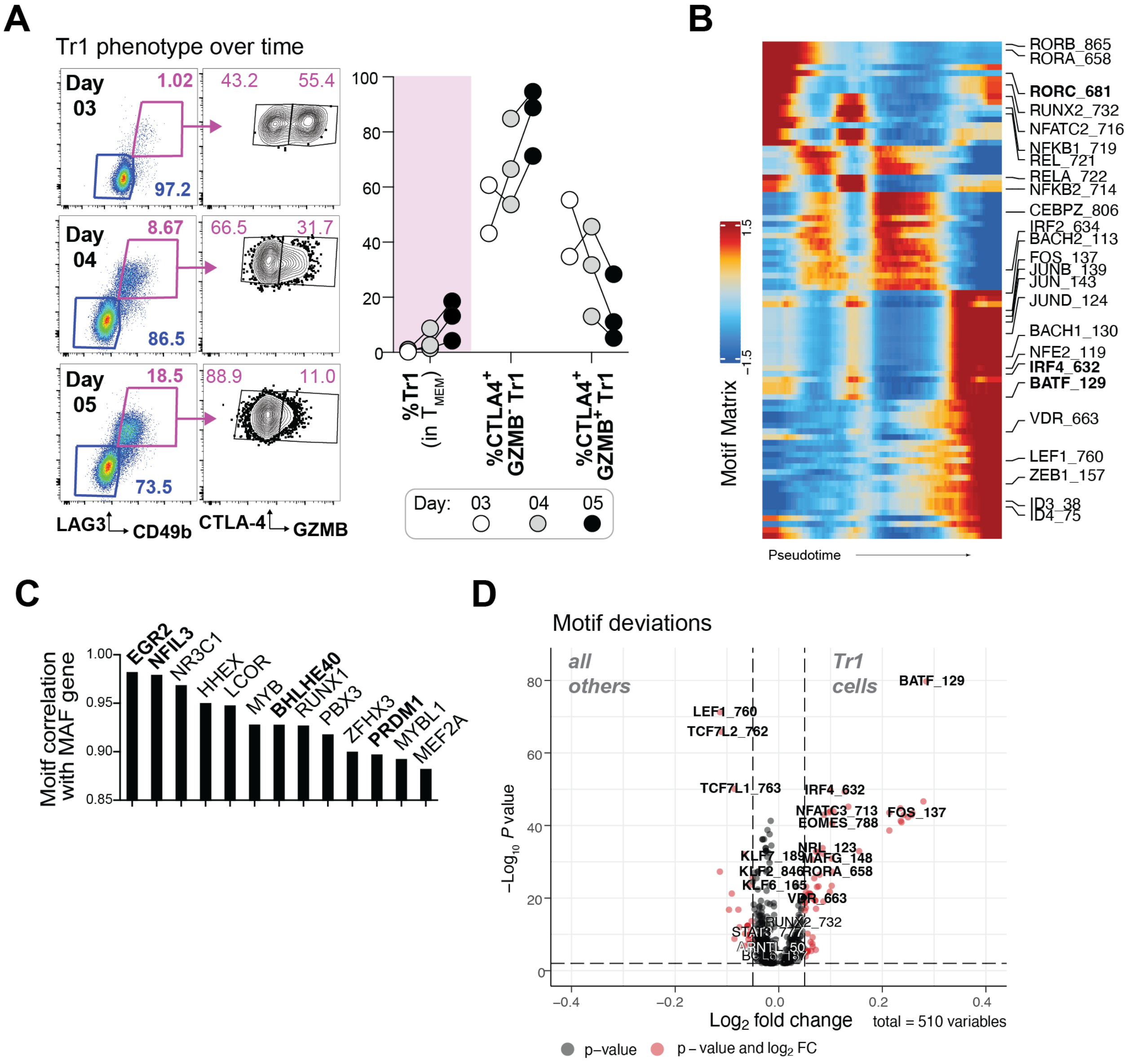


**Fig. S7. A**. Representative flow cytometry plots depicting the analysis of intracellular CTLA-4 and granzyme B (GZMB) expression in CD49b$^+$LAG3$^+$ Tr1 and CD49b$^-$LAG3$^-$ non-Tr1 (DN) memory CD4$^+$ T cells from T-allo10 products over time. Right: cumulative data of Tr1 cell frequency, and intracellular CTLA-4 and GZMB expression in Tr1 cells over time; n = 3. **B.** Heatmap showing transcription factor (TF) motif accessibility dynamics over pseudotime (x-axis = pseudotime bins) across Tr1-only cell clusters, highlighting key motifs that change during Tr1 differentiation (bold). **C.** Bar plot illustrating the correlation between motif accessibility and MAF expression levels, sc-multiome data. In bold: TFs that regulate IL-10 and/or IFN-g. **D.** Volcano plot of differential motif accessibility comparing Tr1 cells to all other CD4$^+$ T cell subsets, sc-multiome dataset.

# Supplementary Figure 8

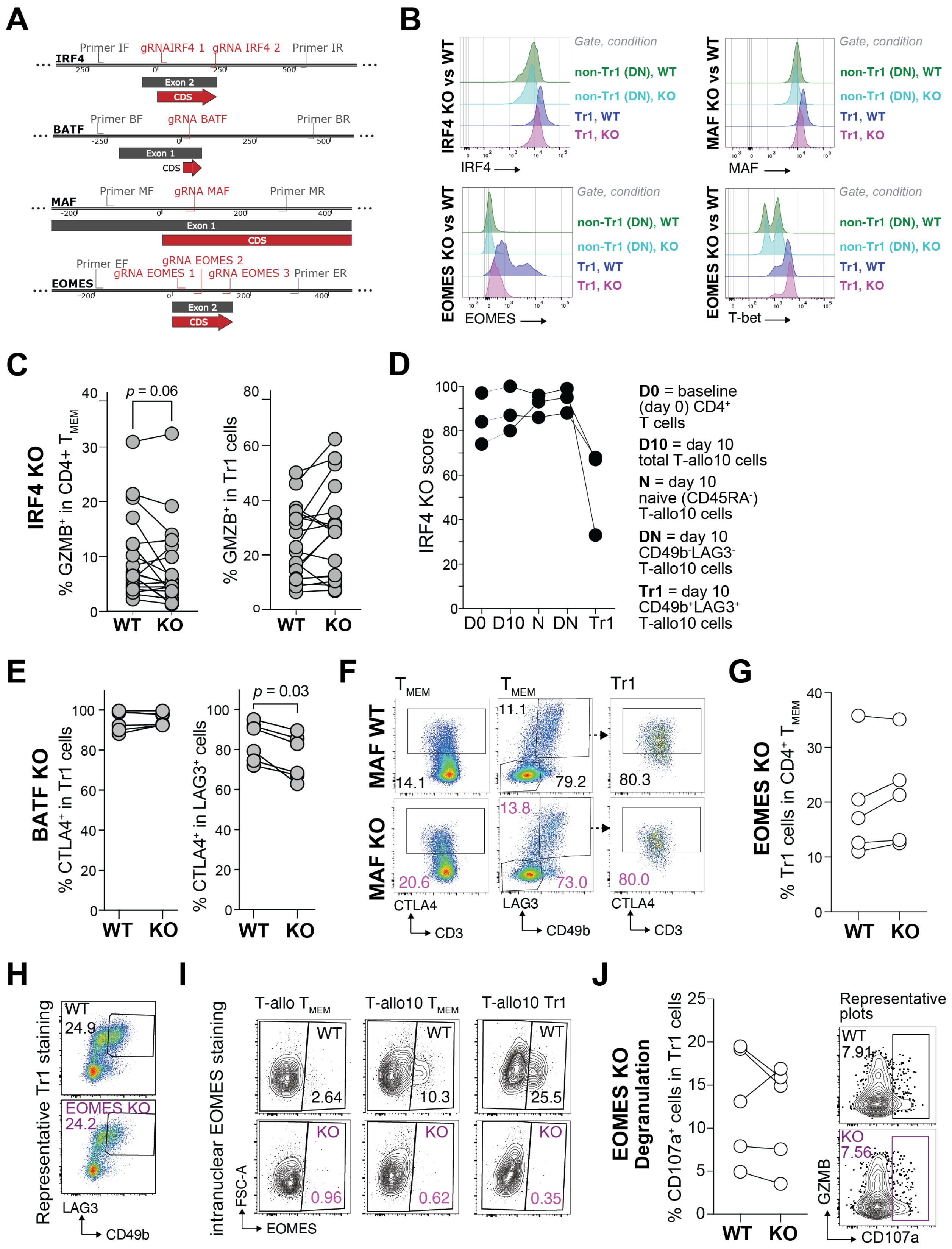

**Fig. S8. A.** CRISPR/Cas9 single guide RNA targeting strategy for IRF4, BATF, MAF, and EOMES, indicating single guide (sg) RNA targets and primers used for PCR. CDS = coding sequence. **B.** Expression of indicated transcription factors (TF) in $CD45RA^{-}CD49b^{+}LAG3^{+}$ Tr1 cells or $CD45RA^{-}CD49b^{-}LAG3^{-}$ non-Tr1 (DN) cells from T-allo10 products. Representative plots of 3 experiments, flow cytometry. **C**. Expression of granzyme B (GZMB) in memory $CD4^{+}$ T cells (Tmem, left) and Tr1 cells (right) in wild-type (WT) and IRF4 knock-out (KO) T-allo10 cells, flow cytometry. **D.** IRF4 KO scores, measured by Synthego ICE (Inference of CRISPR Edits) analysis of Sanger sequencing data, of parental $CD4^{+}$ T cells after 72h rest post-eding (baseline, D0), total T-allo10 cells after 10 day differentiation *in vitro* (D10), and FACS-sorted live singlet $CD4^{+}CD3^{+}$ T cells from T-allo10 cells that were either total $CD45RA^{+}$ (naïve, N), $CD45RA^{-}CD49b^{-}LAG3^{-}$ (DN), or $CD45RA^{-}CD49b^{+}LAG3^{+}$ (Tr1). **E.** Expression of CTLA-4 in $CD49b^{+}LAG3^{+}$ Tr1 cells (Tr1, left) and in $CD49b^{-}LAG3^{+}$ Tmem ($LAG3^{+}$, right) in WT and BATF KO T-allo10 cells, flow cytometry. n = 6. **F**. Representative flow cytometry plots showing frequency of CTLA-$4^{+}$ cells and Tr1 cells in Tmem (left and middle panels, respectively), and CTLA-4 expression within Tr1 cells (right panel), in WT and MAF KO T-allo10 cells. **G**. Frequency of Tr1 cells in EOMES KO or WT T-allo10 cells; flow cytometry. **H**. Representative flow cytometry plots of Tr1 cells in EOMES KO or WT T-allo10 cells. **I**. Representative flow cytometry plots of $EOMES^{+}$ cells in Tr1 and Tmem of EOMES KO or WT T-allo10 cells, compared to Tmem of EOMES KO or WT T-allo cells (control effector T cells). **J**. Left: cumulative flow cytometry data, and right: representative plots, showing the frequency of Tr1 cells expressing surface CD107a, which indicates degranulating cells, after 6h-culture of T-allo10 products in the presence of anti-CD3/CD28 Dynabeads; gate for positive population was based on the CD107a expression in unstimulated condition (not shown). Note: one donor had below average KO efficiency. Statistical analysis, panels C, E, G, J: Wilcoxon test, $p$ = n.s. unless otherwise indicated.

# Supplementary Figure 9

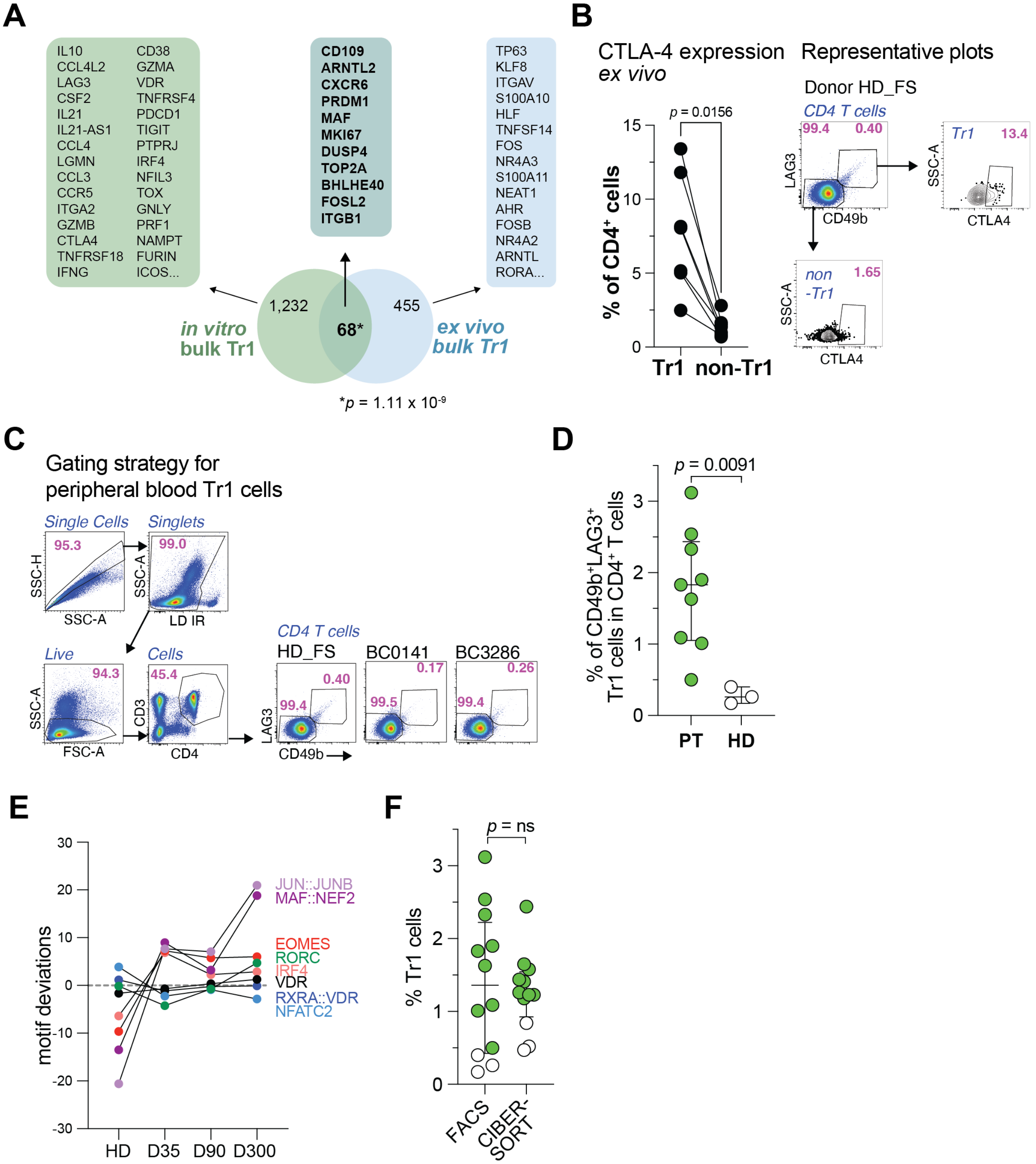

**Fig. S9. A.** Venn diagram illustrating the overlap between significantly upregulated genes in *in vitro*-induced, T-allo10 Tr1 cells (compared to control cells, from Chen P and Cepika AM et al (7); absolute log2 fold-change (absLog2FC) >2, FDR adjusted *p*-value (*p*adj) < 0.05) and *ex vivo* isolated, peripheral blood Tr1 cells (compared to memory $CD4^+$ T cells, from Uyeda MJ and Freeborn R et al(25); absLog2FC > 1.25, *p*adj < 0.05) analyzed by bulk RNA-seq. Boxes contain representative genes. Significance of the overlap was calculated using hypergeometric test. **B.** Expression of intracellular CTLA-4 (n = 7) in *ex vivo* $CD49b^+LAG3^+$ Tr1 and $CD49b^-LAG3^-$ non-Tr1 (DN) cells within total $CD4^+$ T cells from unstimulated PBMC. Left: cumulative data, right: representative flow cytometry gating, healthy donor; Wilcoxon test. **C.** Top: Representative flow cytometry gating strategy to identify Tr1 cells in $CD4^+$ T cells within *ex vivo* PBMC. Bottom right: frequency of Tr1 and non-Tr1 (DN) cell populations in $CD4^+$ T cells of healthy donors used for CIBERSORTx analysis. Gates and donor ID are indicated above the plots. **D.** Flow cytometry analysis of Tr1 frequency within total $CD4^+$ T cells in patient (PT, n = 3; 3 time-points) and healthy donor (HD, n = 3; 1 time-point) samples that were also analyzed by ATAC-seq. Mann-Whitney U test. **E.** Line plot showing accessibility of key transcription factor motifs in ATAC-seq data of peripheral blood $CD4^+$ T cells from healthy donors (HD) and patients treated with T-allo10 cell infusion and allo-HSCT, analyzed at day (D) 35, 90, and 300 post-treatment. **F.** Dot plot showing the percentage of Tr1 cells assessed using two methods: (1) flow cytometry analysis of $CD49b^+LAG3^+$ cells within live $CD4^+$ T cells, and (2) CIBERSORTx deconvolution of gene accessibility data, which show the relative abundance of Tr1 cells in the mixed population. White dots: healthy donors, green dots: patients. Wilcoxon test.

# Supplementary Figure 10

A

B

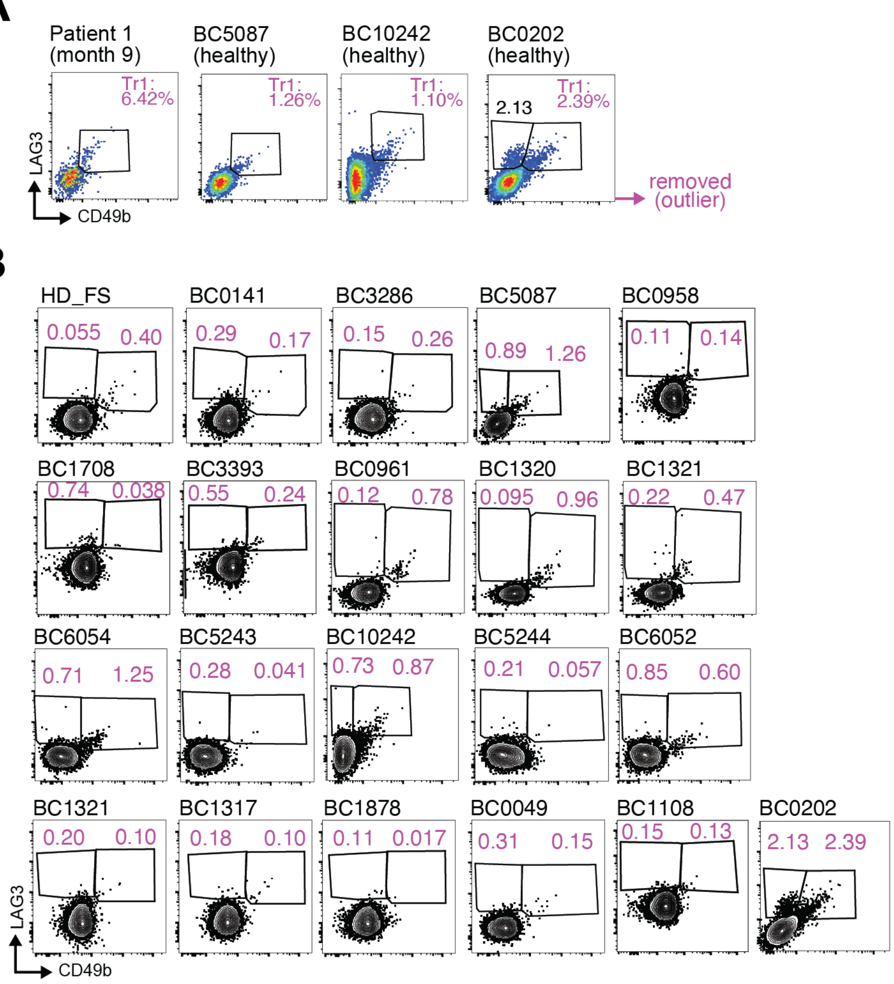


C

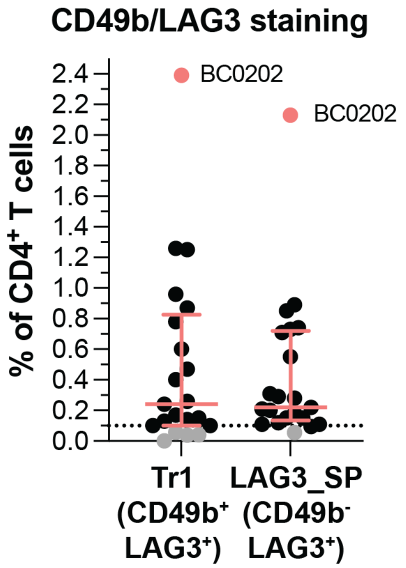


D

## Outlier analysis

| | Tr1 | LAG3_SP |
|---|---|---|
| Method | | |
| ROUT (Q = 1%) | | |
| | | |
| Number of points | | |
| # Y values analyzed | 21 | 21 |
| Outliers | 1 | 1 |

| | Tr1 | LAG3_SP |
|---|---|---|
| Method | | |
| Grubbs (Alpha = 0.05) | G = 3.209 | G = 3.566 |
| | | |
| Number of points | | |
| # Y values analyzed | 21 | 21 |
| Outliers | 1 | 1 |

Outliers in both methods = BC0202 samples

**Fig. S10. A.** Gating strategy to identify CD49b$^+$LAG3$^+$ Tr1 cells in CD4$^+$ T cells from PBMC of healthy donors and patient samples used for sc-multiome. Flow cytometry. **B.** Tr1 frequency in PBMC of additional 20 healthy donors in comparison to donor BC202, which has high population of LAG3-single-positive (SP) CD4$^+$ T cells and elevated Tr1 cells compared to other donors. Flow cytometry. **C.** Cumulative frequency of Tr1 cells and LAG3-SP CD4$^+$ T cells in healthy donor PBMC (n = 21). Line and error bars = median and interquartile range. **D.** Identification of statistical outliers, Robust Regression and Outlier Removal (ROUT) and Grubbs methods.

# Supplementary Figure 11

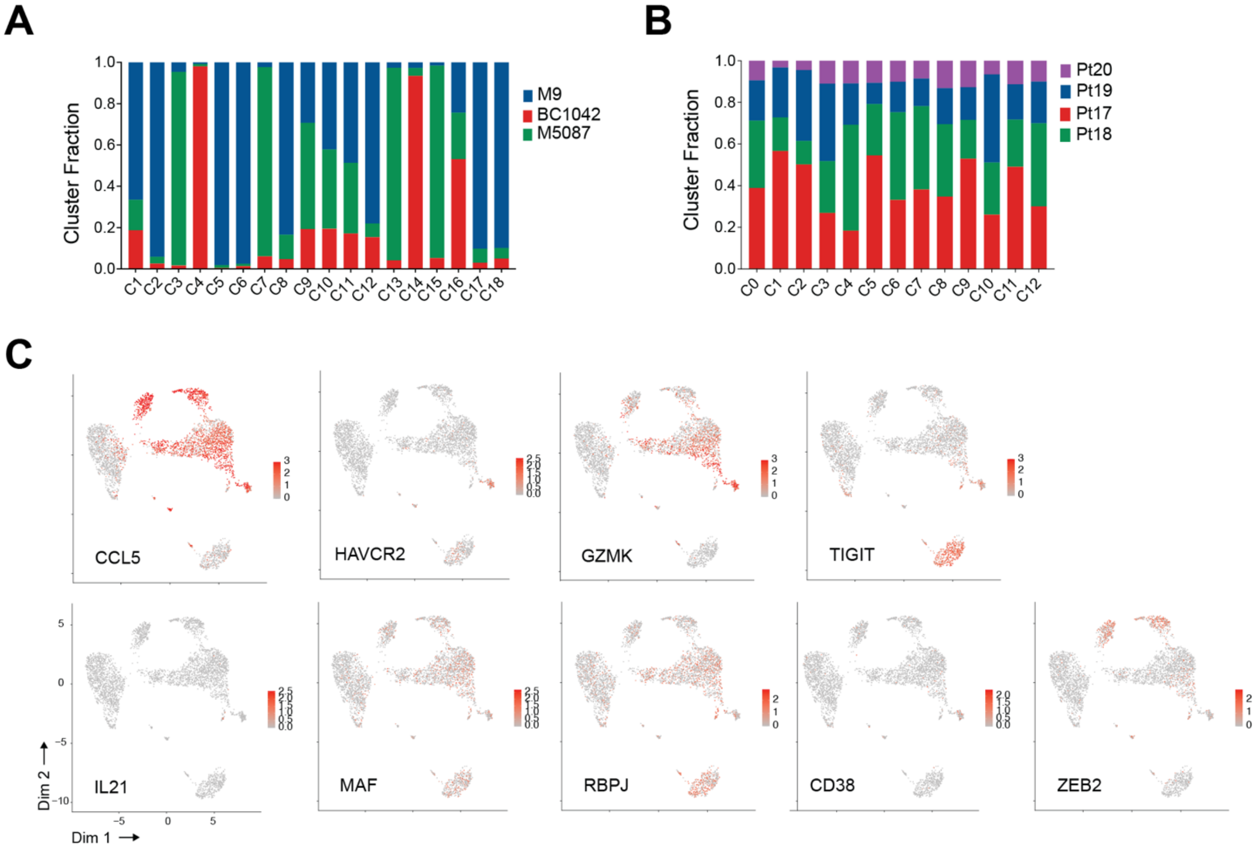


**Fig. S11. A.** Donor distribution across clusters of CD4$^+$ T cells shown in Figure 5E. M9 = Patient treated with T-allo10 infusion and allo-HSCT, analyzed 9 months post-treatment. BC = healthy donors. **B.** Patient (Pt) distribution across CD4$^+$ T cell clusters from Figure 6C, re-analyzed clear-cell renal cell carcinoma (ccRCC) sc-RNA-seq dataset. **C.** UMAP embeddings showing gene expression levels of additional Tr1 markers in the ccRCC sc-RNA-seq dataset.

# Supplementary Figure 12

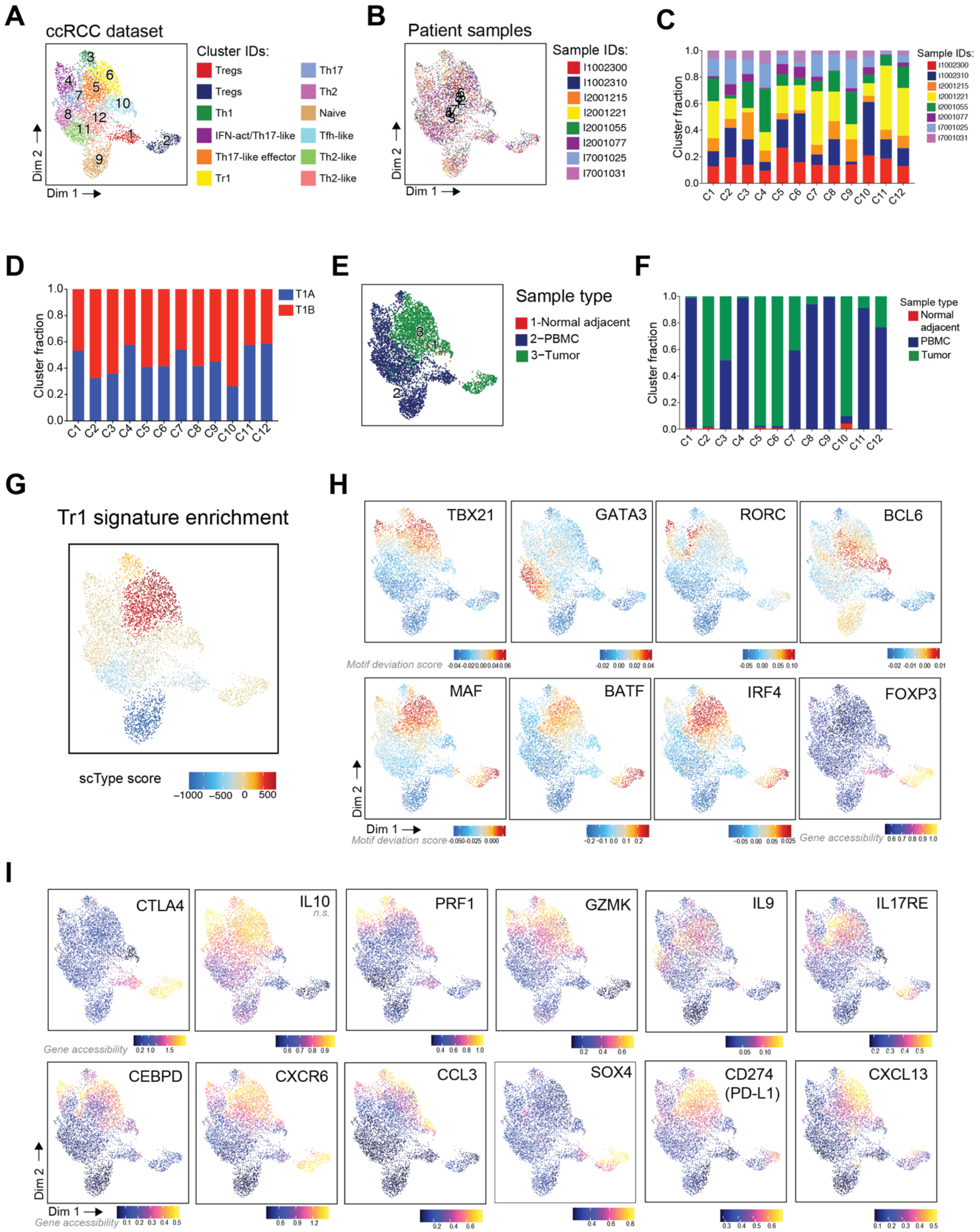

**Fig. S12. A.** Re-analysis of $CD4^+$ T cells from the clear-cell renal cell carcinoma (ccRCC) sc-ATAC-seq dataset, depicting 12 annotated clusters in a UMAP embedding. **B.** UMAP representation of 8 ccRCC samples after integration with Harmony algorithm. Color indicates distinct patient sample IDs. **C.** Fractional distribution of patients across identified clusters. **D.** Fractional distribution of cancer stages (Ia or Ib) within each cluster. **E.** UMAP representation highlighting cell origins; tumor = cells isolated from kidney tumor tissue; normal adjacent = cells isolated from the adjacent normal kidney tissue; PBMC = cells isolated from peripheral blood mononuclear cells. **F.** Fractional distribution of cell origins within each cluster. **G.** Enrichment of the Tr1-specific gene expression signature. Color scale = scType score. **H, I.** UMAP embedding of accessible transcription factor motifs (chromVAR deviation scores (H)) or accessible genes (gene score accessibility (I)), as visualized by ArchR for indicated genes.

# Supplementary Figure 13

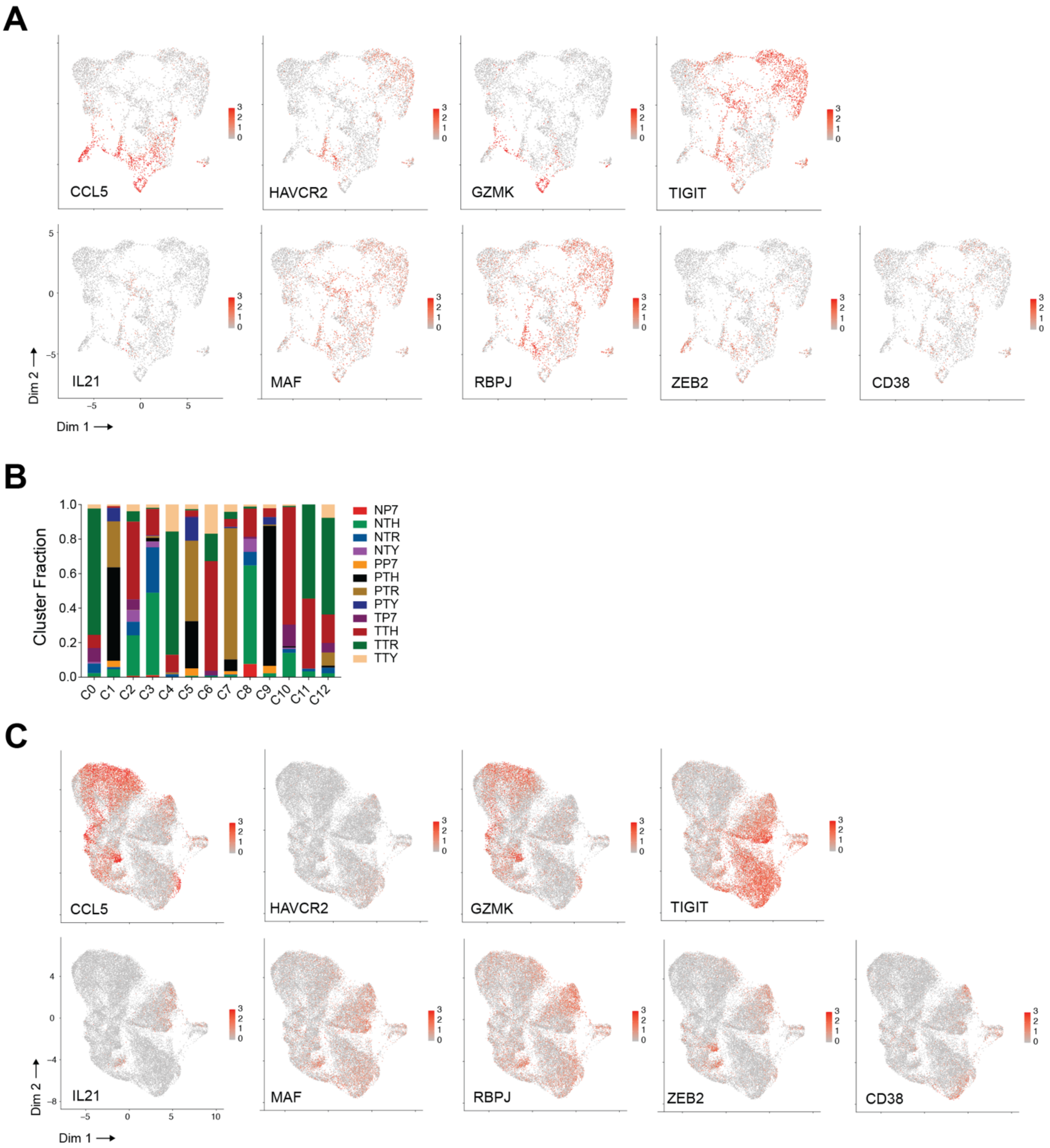

**Fig. S13. A.** UMAP embeddings showing gene expression of additional Tr1 markers in the re-analyzed colorectal cancer (CRC) sc-RNA-seq dataset. **B.** Distribution of tissue type across clusters from the CRC dataset. C6 is the predicted Tr1 cluster. Abbreviations: PTY = peripheral blood CD4 ($CD25^{int}$), NTY = adjacent normal CD4 ($CD25^{int}$), TTY = tumor CD4 ($CD25^{int}$) , PTH = peripheral blood Th CD4 ($CD25^{-}$), PP7 = peripheral blood CD4, NTH = adjacent normal Th CD4 ($CD25^{-}$) , NP7 = normal adjacent CD4, TTH = tumor Th CD4 ($CD25^{-}$) TP7 = tumor CD4 , PTR = peripheral blood Treg ($CD25^{++}$), NTR = adjacent normal Treg ($CD25^{++}$), TTR = tumor Treg ($CD25^{++}$). **C**. UMAP embeddings showing gene expression of additional Tr1 markers across tissue $CD4^{+}$ T cells in the re-analyzed triple-negative breast cancer (TNBC) sc-RNA-seq dataset.

# Supplementary Figure 14

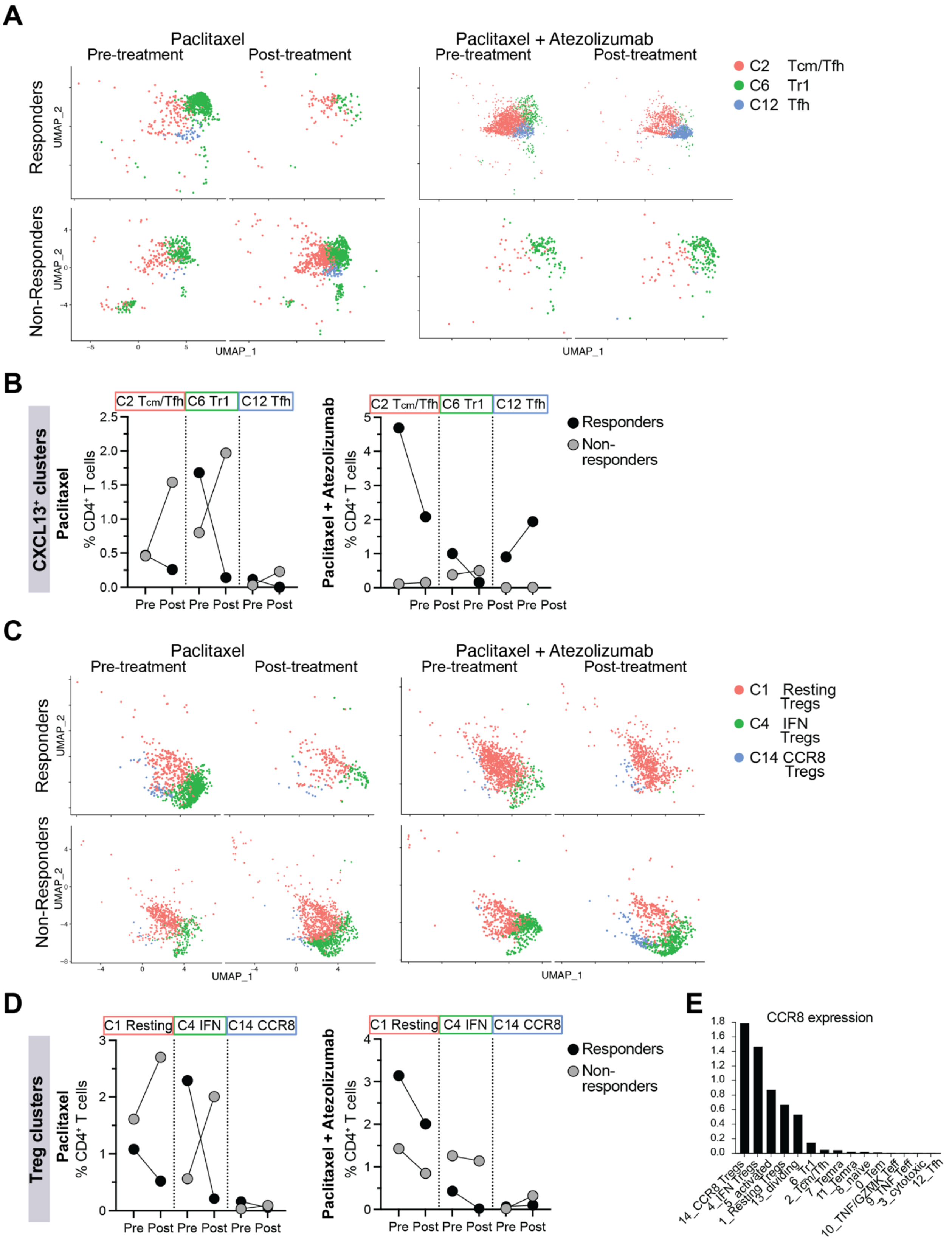

**Fig. S14. A, C.** UMAP representation of $CD4^+$ $CXCL13^+$ T clusters (A) or $FOXP3^+$ Treg clusters (C) showing temporal alterations in their cell proportions between responders and non-responders to chemotherapy (paclitaxel) or combination therapy (paclitaxed and atezolizumab, anti-PD-L1) in the re-analysis of the published triple-negative breast cancer (TNBC) sc-RNA-seq dataset. **B, D.** Frequency of cells in CXCL13+ T cell (B) and FOXP3+ Treg (D) clusters among total CD4+ T cells isolated from responder and non-responder TNBC patients pre- and post-treatment. **E.** CCR8 expression level in TNBC dataset clusters.

# Supplementary Figure 15

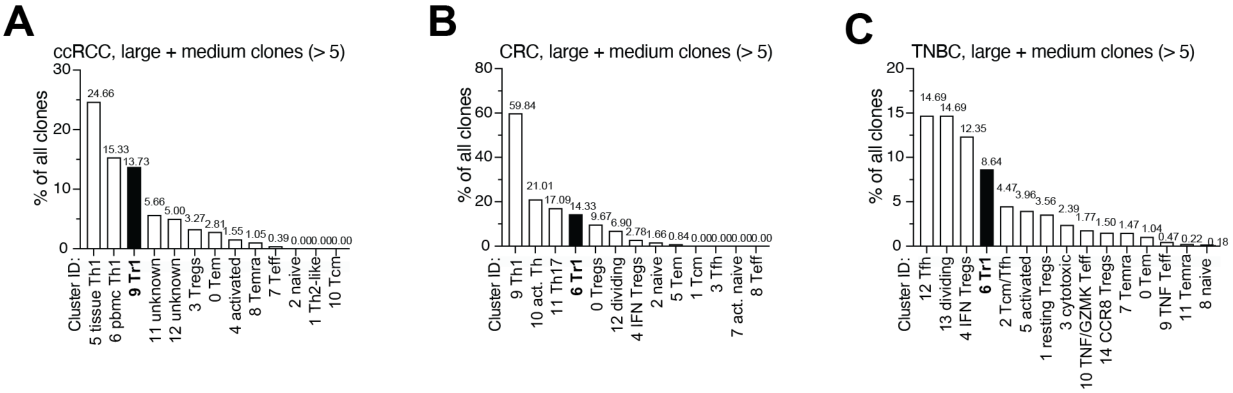


**Fig. S15.** **A**, **B**, **C**. Proportion of identified large and medium clones (> 5 cells) per cluster of $CD4^+$ T cells; matched sc-TCR-seq data of the re-analyzed clear-cell renal cell carcinoma (ccRCC) dataset, colorectal cancer dataset (CRC), and triple-negative breast cancer dataset (TNBC), respectively.

**Data S1. (separate file)**
Supplementary Tables 1-20.

**Data S2. (separate file)**
Source data for *in vitro* experiments with small sample size.
